# A human blood-brain barrier model reveals pericytes as critical regulators of viral neuroinvasion

**DOI:** 10.1016/j.isci.2025.114443

**Published:** 2025-12-13

**Authors:** Alexsia Richards, Andrew Khalil, Punam Bisht, Troy W. Whitfield, Xinlei Gao, David Mooney, Lee Gehrke, Rudolf Jaenisch

**Affiliations:** 1 Whitehead Institute for Biomedical Research, Cambridge, MA 02142, USA; 2 Wyss Institute for Biologically Inspired Engineering, Harvard University, Boston, MA 02115, USA; 3 John A. Paulson School of Engineering and Applied Sciences, Harvard University, Cambridge, MA 02138, USA; 4 Department of Microbiology, Harvard Medical School, Boston, MA 02115, USA; 5 Institute for Medical Engineering and Science, Massachusetts Institute of Technology, Cambridge, MA 02139, USA; 6 Department of Biology, Massachusetts Institute of Technology, Cambridge, MA 02139, USA

**Keywords:** Neuroscience, Cell biology

## Abstract

The blood-brain barrier (BBB) plays a vital role in regulating the passage of biomolecules between the bloodstream and the central nervous system (CNS) while also protecting the CNS from pathogens. Pericytes reside at the interface between endothelial cells and the brain parenchyma. These cells are critical for maintaining BBB integrity and regulating vessel permeability, blood flow, and immune cell migration. In this study, we developed a serum-free protocol to generate neural crest cell-derived pericytes (NCC-PCs) from human pluripotent stem cells (hPSCs). These NCC-PCs can be co-cultured with hPSC-derived brain microvascular endothelial cells (BMECs) in a co-culture BBB model that recapitulates the *in vivo* cellular interactions at the BBB. We used this model to evaluate the pathological consequences of BBB exposure to highly neuroinvasive flaviviruses. Our results identify a previously undescribed role for NCC-PCs in maintaining BMEC barrier integrity during infection and reducing the spread of viral infection to the CNS.

## Highlights

• Neural crest-derived pericytes are generated from stem cells by a serum-free protocol
• Exposure to pericytes reduces breakdown of the endothelial barrier following infection
• Pericytes show polarized release of virus when plated in a Transwell system

## Graphical abstract

**Figure:**
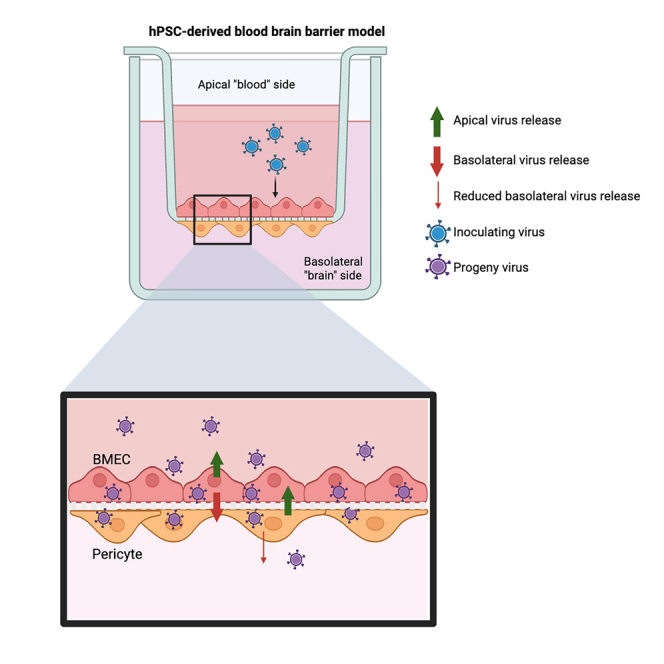

## Introduction

Neuroinvasive flaviviruses, including West Nile virus (WNV), Powassan virus (POWV), and Japanese encephalitis virus (JEV), pose significant public health threats due to their ability to infect the central nervous system, leading to severe neurological complications.^1^ The blood-brain barrier (BBB) is thought to be one of the key access points for these viruses to reach the CNS.^2^^,^^3^ Evidence from *in vitro* models has generated multiple hypotheses on how these viruses cross the BBB, including transcytosis through infected endothelial cells, paracellular transport through the intercellular space between cells, disruption of the barrier, and transport via infection of migrating immune cells.^3^^,^^4^^,^^5^

The BBB is a critical interface that tightly regulates the passage of biomolecules between the bloodstream and the central nervous system (CNS), while also protecting the CNS from invading pathogens. Pericytes (PCs) are multifunctional mural cells that wrap around the endothelial cells of capillaries and venules throughout the body.^6^^,^^7^^,^^8^ At the BBB, the interaction between PCs and brain microvascular endothelial cells (BMECs) is crucial for regulating vessel permeability.^9^^,^^10^ Additionally, PCs can exhibit macrophage-like phagocytic phenotypes, contributing to the clearance of circulating toxins, and regulate the migration of immune cells across the BBB.^11^^,^^12^^,^^13^

Studying the mechanisms by which neuroinvasive viruses cross the BBB is difficult *in vivo* and has long been done with *in vitro* models. However, many *in vitro* models using primary cells often fail to incorporate multiple BBB cell types and exhibit poor endothelial barrier function, making it difficult to extrapolate these findings to *in vivo* pathology. Advancements in stem cell technology have improved *in vitro* BBB models by enabling the production of isogenic populations of nearly all BBB cell types.^14^ During development, forebrain pericytes arise from neural crest cells (NCCs).^15^^,^^16^ Although protocols have been published for the differentiation of neural crest-derived pericytes (NCC-PCs) from human pluripotent stem cells (hPSCs),^17^^,^^18^ these protocols utilize fetal bovine serum (FBS) in the differentiation media. In addition to its intrinsic variability,^19^^,^^20^^,^^21^ FBS is known to activate inflammatory signaling and reduce epithelial barrier function, which may present challenges when incorporating these protocols with additional hPSC-derived BBB cell types.^22^^,^^23^ Serum can also inhibit infection with some viruses, further complicating studies on viral infection in these cells.^24^^,^^25^^,^^26^ To address these potential issues, we developed a protocol for generating hPSC-derived NCC-PCs using fully defined, serum-free media for both differentiation and maintenance. We further integrated these NCC-PCs with BMECs to create an *in vitro* co-culture BBB model.

Using our hPSC-derived BBB model, we demonstrate that neuroinvasive flaviviruses infect multiple BBB cell types, while exerting divergent effects on BMEC barrier function that correlate with *in vivo* disease severity. Additionally, we identify a previously undescribed protective role for NCC-PCs during WNV infection at the BBB.

## Results

### Serum-free differentiation of neural crest-derived pericytes from human pluripotent stem cells

**Figure 1:**
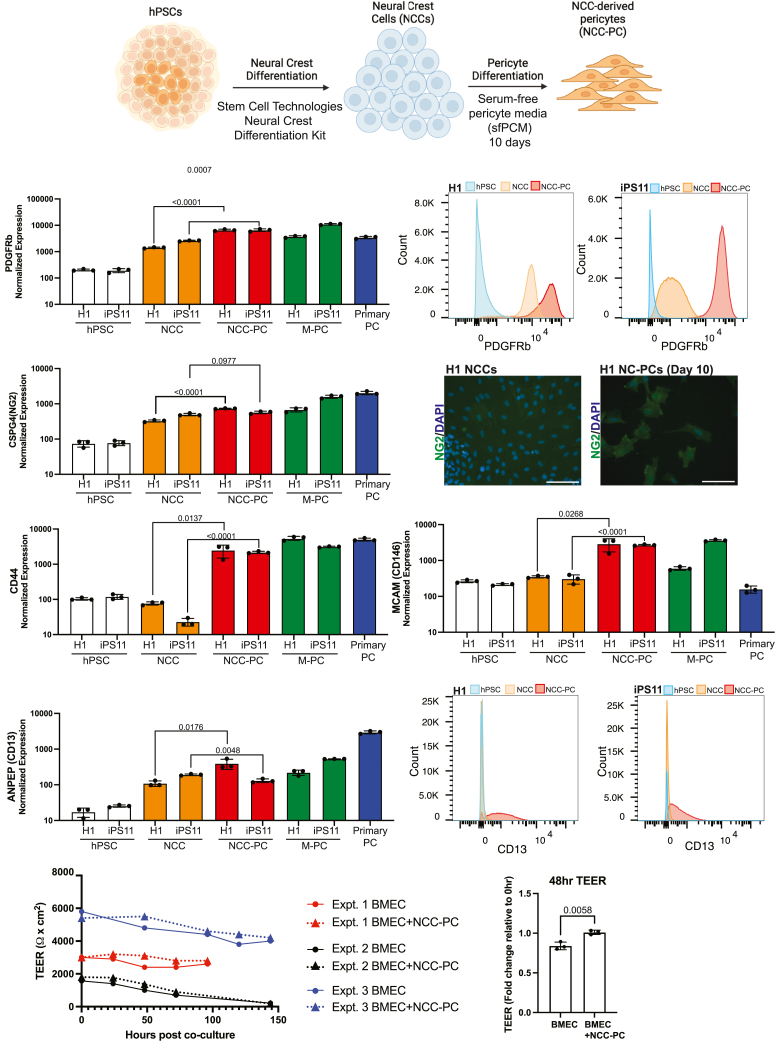
Generation and characterization of hPSC-derived neural crest pericytes (A) Schematic of differentiation of neural crest pericytes (NCC-PCs) from hPSCs. (B, D, F–H) Bulk RNA sequencing was performed on hPSCs, hPSC-derived neural crest cells (NCC), hPSC-derived NCC-PCs (NCC-PC), hPSC-derived mesoderm pericytes (M-PC), or primary CNS pericytes (Primary PC). H1 or iPS11 stem cells were used for all differentiations. All bar graphs show the mean value of three biological replicates; error bars show standard deviation. (B) Normalized RNA expression of PDGFRb. (C) Expression of PDGFRb quantified by flow cytometry on H1 or iPS11 hPSCs, NCCs, and NCC-PCs. (D) Normalized RNA expression of CSPG4 (NG2). (E) Immunofluorescence images of H1 hPSC-derived NCCs or H1 hPSC-derived NCC-PCs stained with an antibody directed against NG2. Scale bars = 100um. (F) Normalized RNA expression of CD44 (G), normalized RNA expression of CD146, and (H) normalized RNA expression of CD13. (I) Expression of CD13 quantified by flow cytometry on H1 or iPS11 hPSCs, NCCs, and NCC-PCs. (J) TEER values of H1 hPSC-derived BMEC-like cells plated in Transwell plates with or without H1 hPSC-derived NCC-PCs plated in the lower chamber. TEER was measured beginning at 0 h after the initiation of co-culture. TEER values were measured in three independent experiments. The fold change in TEER values from 0 to 48 h after the initiation of co-culture was quantified for each experiment and compared using an unpaired *t* test.

We established a two-step protocol to generate and maintain neural crest-derived PCs derived from hPSCs (Figure 1A). Similar to previously published protocols, our approach begins with the differentiation of NCCs from hPSCs^18^^,^^27^ using a commercial kit (Stem Cell Technologies) following the manufacturer’s protocol. Following differentiation, NCCs were cultured in serum-free pericyte media (sfPCM) for ten days to generate NCC-PCs. We have previously shown that sfPCM supports the differentiation and maintenance of mesoderm-derived pericytes.^28^ To confirm that our protocol worked with multiple hPSC lines, we performed differentiations with both H1 embryonic stem cells and iPS11 induced pluripotent stem cells. Defining pericyte-specific marker proteins remains challenging as the expression of many proteins overlaps with other mural cells and varies depending on developmental stage, location, and experimental conditions. Therefore, a combination of expression of multiple marker proteins should be considered in the characterization of pericytes. We examined the expression of key marker genes at each step of our protocol by bulk RNA sequencing, specifically comparing expression in hPSCs, hPSC-derived NCCs, and day 10 hPSC-derived NCC-PCs (Figure 1A). We included both primary human brain PCs as well as hPSC-derived mesoderm PCs as controls for PC gene expression. As expected, only hPSCs expressed a significant amount of the transcription factors POU5F1 and NANOG (Figures S1A and S1B). NCCs showed significantly higher levels of the neural crest markers SOX9^29^ and TFAP2A^30^^,^^31^ and NGFR^32^ relative to hPSCs (Figures S1C–S1E). To confirm that we had generated neural crest cells, we stained NCCs with an antibody directed against the neural crest marker Sox10.^33^ Our results showed that Sox10 was expressed in our hPSC-derived NCCs (Figure S1F). To quantify the purity of our neural crest population, we performed FACS analysis using an antibody directed against Sox10. Our results show that greater than 99% of our neural crest cells express Sox10 relative to the unstained control neural crest cells (Figure S1G).

The co-expression of platelet-derived growth factor receptor beta (PDGFRb) and chondroitin sulfate proteoglycan 4 (CSPG4) is considered a hallmark of pericytes.^34^ Our results showed that, at both the RNA and protein level, expression of PDGFRb was significantly higher in our hPSC-derived NCC-PCs when compared to hPSCs or hPSC-derived NCCs (Figures 1B and 1C). Surprisingly, our hPSC-derived NCC-PCs showed higher expression, at the RNA level, of PDGFRb than primary brain pericytes. Studies have shown that PDGFRb is more highly expressed in immature pericytes;^35^ therefore, it is possible that our hPSC-derived pericytes represent a less mature population than primary brain pericytes. CSPG4, also known as neural glial antigen 2 (NG2), is a well-established pericyte marker.^36^^,^^37^^,^^38^ Both H1 and iPS11 NCC-PCs showed significant upregulation of NG2 relative to hPSCs, but only H1 NCC-PCs showed significant upregulation relative to the intermediate hPSC-derived NCCs (Figure 1D). We further examined NG2 protein expression by immunofluorescence in hPSC-derived NCCs and NCC-PCs using an antibody directed against NG2. Our hPSC-derived NCC-PCs showed increased NG2 staining compared to NCCs (Figure 1E).

The cell surface proteins CD44, CD146, and CD13 are expressed by pericytes, with CD146 playing a key role in the PDGFRb-mediated recruitment of pericytes.^6^^,^^39^^,^^40^^,^^41^^,^^42^ Our hPSC-derived NCC-PCs expressed CD44 and CD146 at significantly higher levels than hPSCs or hPSC-derived NCCs (Figures 1F and 1G). CD13 RNA levels were also slightly elevated in hPSC-derived NCC-PCs relative to hPSC-derived NCCs (Figure 1H). We quantified CD13 surface expression by FACS and observed higher levels in both H1 and iPS11 NCC-PCs relative to hPSCs or hPSC-derived NCCs (Figure 1I).

The transcription factors FoxF2 and FoxC1 are expressed in pericytes early in development and are essential for the development and maintenance of the BBB in mouse models.^43^^,^^44^^,^^45^ We observed low levels of expression of FoxF2 in our hPSC-derived NCC-PCs and mesoderm-derived PCs (Figure S2A). This result is consistent with previous reports on hPSC-derived PCs showing low expression of this transcription factor.^18^ One hypothesis is that the expression of FoxF2 may be tightly regulated and not detectable at the time point examined or requires signaling from other BBB cell types to increase expression. Our hPSC-derived NCC-PCs did show increased expression, at the RNA level, of the FoxC1 transcription factor (Figure S2B).

Smooth muscle actin (ACTA) and the actin-binding proteins Calponin (CNN1) and Transgelin (TAGLN) are expressed by multiple types of mural cells, including smooth muscle cells and subsets of pericytes.^46^^,^^47^^,^^48^^,^^49^ Our hPSC-derived NCC-PCs showed higher levels of calponin relative to hPSCs or NCCs (Figures S2C and S2D). ACTA expression was not significantly increased relative to hPSC-derived NCCs in either cell line, and only iPS11-derived NCC-PCs showed significant upregulation of TAGLN relative to NCCs (Figures S2E and S2F). Notably, ACTA was higher in our hPSC-derived mesoderm PCs, suggesting that these cells may share more transcriptional overlap with smooth muscle cells, which share a mesoderm origin. Finally, angiopoietin-1 (ANGPT1) is a secreted glycoprotein expressed by pericytes and smooth muscle cells that is critical for endothelial cell survival and angiogenesis.^50^^,^^51^^,^^52^ Our hPSC-derived NCC-PCs expressed higher levels of ANGPT1 than hPSCs or hPSC-derived NCCs (Figure S2G).

To further characterize our hPSC-derived NCC-PCs, we performed principal component analysis (PCA) on counts data from RNA-seq experiments using hPSC-derived NCC-PCs, hPSC-derived mesoderm PCs, and primary brain PCs (Figure S3). In the PCA plot, the first principal component (PC1) captures nearly 41% of the variance, and the second principal component (PC2) captures 20%. The first principal component delineates the initial hPSC cells (i.e., H1 hESCs or iPS11 iPSCs) from the differentiated cells, while the second principal component delineates hPSC-derived NCCs from hPSC-derived pericytes and primary brain pericytes.

We also performed a transcriptomic comparison of our NCC-PCs to previously published single-cell RNA sequencing data from *in vivo* pericytes^53^ (Figures S4A and S4B). We observe moderately high Pearson’s correlation coefficients of 0.78 for H1 NCC-PCs (Figure S4A) and 0.77 for iPS11 NCC-PCs (Figure S4B). Critically, this degree of correlation was similar to that observed in a comparison of bulk RNA sequencing data from cultured primary brain pericytes to the single-cell sequencing data from *in vivo* pericytes (*r* = 0.79, Figure S4C), suggesting that our hPSC-derived NCC-PCs are as similar to *in vivo* pericytes as are cultured primary brain pericytes. In addition, we note that the expression of PDGFRB, a key factor in the growth and migration of pericytes, aligns better with *in vivo* PCs in our NCC-derived PCs than it does in cultured primary brain pericytes.

The co-culture of brain microvascular endothelial cells (BMECs) with PCs is known to improve barrier function.^27^ Transendothelial electrical resistance (TEER) provides a quantitative readout of endothelial cell barrier function.^54^ We employed a previously published protocol to generate hPSC-derived BMECs, specifically, we used a protocol based on the methods described in Lippman et al.^55^ and Neal et al.^56^ Several studies have shown that hPSC-derived BMECs produced by this protocol have elevated levels of epithelial transcripts relative to primary endothelial cells, therefor caution should be used when attributing the observed phenotypes to being specific to endothelial cells.^57^^,^^58^^,^^59^ However, hPSC-derived BMECs generated using this protocol have been shown to recapitulate BBB function and respond more effectively to co-culture with other BBB cells than other hPSC-derived endothelial cells.^60^ In addition, hPSC-derived BMECs produced via this method have been used in previous studies on flavivirus BMEC infection.^61^ To avoid confusion with true BMECs, we refer to the cells generated by this protocol as hPSC-derived BMEC-like cells (hBMECs), which is consistent with previously published reports.^59^^,^^62^ Following differentiation, we confirmed that our hBMECs expressed the endothelial marker VE-Cadherin (Figure S5A).

To determine if the co-culture of NCC-PCs with hBMECs could improve their barrier function, hPSC-derived hBMECs were plated in the apical chamber of a Transwell plate and NCC-PCs in the lower chamber. TEER values were measured every 24 h for up to 144 h after the initiation of co-culture. Our results show that co-culture with NCC-PCs increased hBMEC TEER values at all time points examined (Figure 1J) and was statistically significant by 48 h after the initiation of co-culture. Consistent with previous studies using hPSC-derived hBMECs, we observed significant variability in the TEER values observed across independent experiments, and in all experiments, TEER values dropped over prolonged time in culture.^56^^,^^63^

Studies have shown that FBS can reduce the barrier function of epithelial cells.^23^ To test if the presence of FBS impaired the barrier function of our hPSC-derived BMEC-like cells, we plated hBMECs on Transwells and replaced the media on the underside of the Transwell with either sfPCM or sfPCM with 1% FBS. TEER values were measured 48 h after media replacement. Our results show that the presence of FBS significantly reduced hBMEC TEER values (Figure S5B).

In conclusion, our data show that functional NCC-PCs can be derived from hPSCs using fully defined serum-free conditions. This protocol provides a viable alternative means of producing neural crest-derived pericytes from pluripotent stem cells that can be used when the incorporation of serum is detrimental to the experimental setup.

### Neuroinvasive flaviviruses infect human pluripotent stem cell-derived blood-brain barrier cells

**Figure 2:**
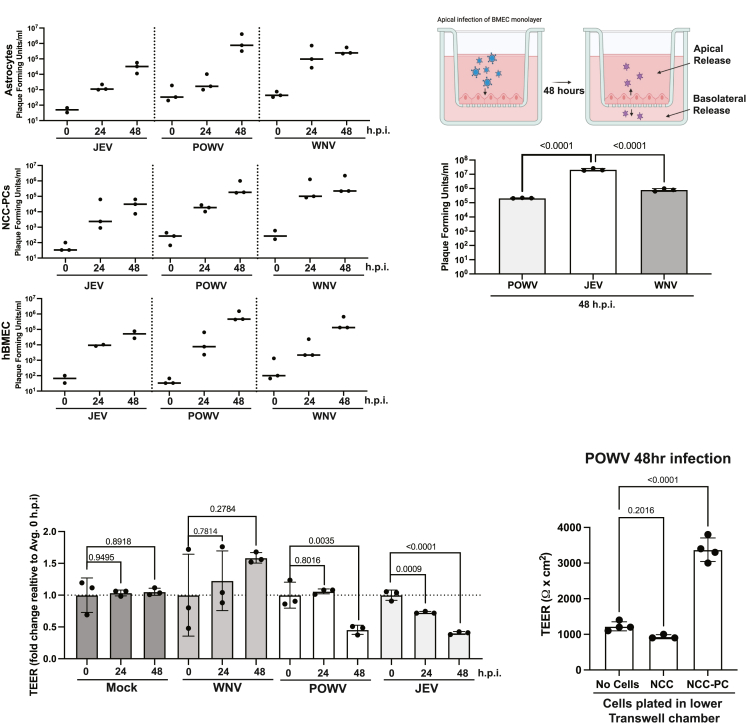
Neurotropic flaviviruses productively infect hPSC-derived blood-brain barrier cells (A) hPSC-derived astrocytes, hPSC-derived NCC-PCs, or hPSC-derived BMEC-like cells (hBMECs) were infected with either JEV, POWV, or WNV at an MOI of 0.1, and the amount of infectious virus released into the media at the indicated time post-infection was quantified by plaque assay. Each data point is the result of an independent experiment. (B) hBMECs were plated in Transwell plates and apically infected with WNV, POWV, or JEV (MOI = 0.1). The amount of infectious virus released into the basolateral chamber of the Transwell was quantified by plaque assay and compared using a one-way ANOVA with Dunnett’s multiple comparisons test, with a single pooled variance. Each data point represents an independent biological replicate. Bar graphs show the mean value, and error bars show the standard deviation. (C) hBMECs were plated in Transwell plates, and TEER was measured at the indicated time after either mock infection or infection with WNV, POWV, or JEV. TEER values are plotted as a fold-change relative to 0 h post-infection and compared using a one-way ANOVA with Dunnett’s multiple comparisons test, with a single pooled variance. Data points represent independent biological replicates. Bar graphs show the mean value, and error bars show the standard deviation. (D) hBMECs were plated in Transwell plates with either hPSC-derived NCCs or hPSC-derived NCC-PCs plated in the lower chamber. hBMECs were infected with POWV 24 h after the initiation of co-culture, and TEER was measured at 48 h after infection. Conditions were compared using a one-way ANOVA with Dunnett’s multiple comparisons test, with a single pooled variance. Each data point represents an independent biological replicate. Bar graphs show the mean value, and error bars show the standard deviation.

Previous studies have shown that BBB cells, including BMECs, astrocytes, and pericytes, can be infected by neuroinvasive flaviviruses;^5^^,^^64^^,^^65^^,^^66^ however, the use of primary cells from different sources and species can make comparisons of the level of infectivity and pathological consequences of infection difficult to interpret. A more recent study demonstrated that flaviviruses can infect hPSC-derived BMECs; however, this study did not examine the infection of other hPSC-derived BBB cell types.^61^ To determine if our hPSC-derived BBB cells can be productively infected by neuroinvasive flaviviruses, hPSC-derived hBMECs, hPSC-derived astrocytes, and hPSC-derived NCC-PCs were infected with WNV, POWV, or JEV at a multiplicity of infection (MOI) of 0.1. The amount of infectious virus released into the media was quantified at 0 h, 24 h, or 48 h post-infection. We observed the robust replication of all three viruses in all three hPSC-derived cell types (Figure 2A). One possible mechanism of spread of infection through the BBB is via basolateral release from infected BMECs to the surrounding perivascular CNS cells. To determine if infected hBMECs released virus to the basolateral chamber following apical infection, hBMECs were cultured in Transwell plates and infected apically with WNV, JEV, or POWV. The amount of infectious virus released into the lower chamber was quantified at 24 and 48 h (Figure 2B). Although infection with all three viruses resulted in basolateral release of nascent virus to the lower chamber, POWV infection resulted in significantly more infectious virus in the lower chamber at 48 h relative to WNV or JEV (Figure 2B).

We next examined hBMEC TEER at 0, 24, or 48 h post-infection. WNV infection did not reduce hBMEC barrier integrity relative to mock-infected hBMECs (Figure 2C). These findings align with previous studies in primary cells showing that WNV infection does not impair endothelial barrier function and may even enhance it compared to mock-infected controls.^64^^,^^67^ Conversely, beginning at 24 h post-infection with JEV or 48 h after infection with POWV, we observed a significant reduction in hBMEC TEER (Figure 2C). Our data showed that co-culture of NCC-PCs with hBMECs improved barrier function (Figure 1F). To determine if co-culture with NCC-PCs could improve hBMEC barrier function following POWV infection, hBMECs were plated in the apical chamber of a Transwell plate, and NCC-PCs or their precursor NCCs were plated in the lower chamber. hBMECs were infected with POWV 24 h after the initiation of co-culture, and TEER was measured at 48 h post-infection. The co-culture of hBMECs with NCC-PCs but not their precursor NCCs resulted in improved hBMEC barrier function following a 48-h POWV infection (Figure 2D).

In summary, our data show that our hPSC-derived BBB cells can be infected with multiple neuroinvasive flaviviruses, and infected hBMECs release nascent virus both apically and basolaterally. Collectively, these data support the hypothesis that the direct infection of BMECs, pericytes, and astrocytes is a viable route for these viruses to enter the CNS. In addition, our data show that POWV and JEV may have a more severe impact on BMEC barrier integrity compared to WNV.

### A co-culture blood-brain barrier model reveals a role for pericytes in reducing West Nile virus neuroinvasion

**Figure 3:**
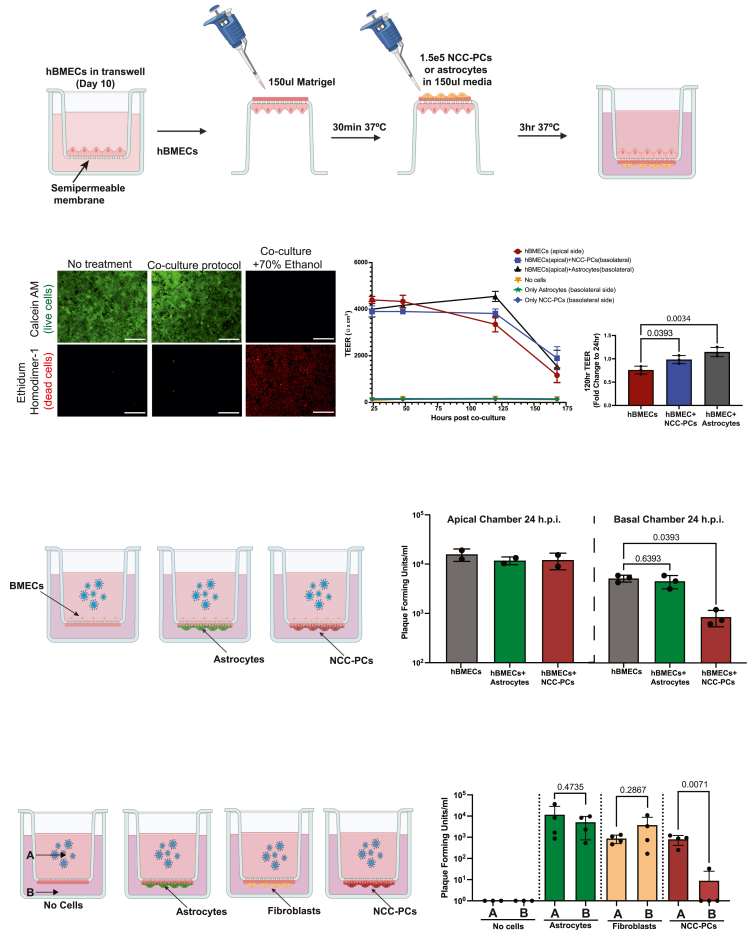
Generation and infection of an hPSC-derived co-culture blood-brain barrier model (hcBBB) (A) Schematic of the generation of hPSC-derived co-culture blood-brain barrier model (hcBBB). (B) hBMECs were simultaneously stained with green-fluorescent calcein-AM to indicate intracellular esterase activity and red-fluorescent ethidium homodimer-1 to indicate the loss of plasma membrane integrity. The loss of green fluorescent signal in combination with red nuclear staining indicates cell death. Viability was compared between hBMECs in the Transwell system without undergoing the co-culture protocol (no treatment) and hBMECs after the co-culture protocol, which included a 30-min incubation with Matrigel and 3 h inverted incubation at 37°C (co-culture protocol). As a positive control, one set of hBMECs underwent the co-culture protocol and were then treated with 70% ethanol to induce cell death (co-culture +70% ethanol). Scale bars = 100um. (C) TEER was measured across the Transwell semipermeable membrane of the hcBBB system beginning 24 h after the initiation of co-culture. Data points show the average and the standard deviation of three independent biological replicates. The change in TEER from 24 h to 120 h after the initiation of co-culture was quantified, and the conditions were compared using a one-way ANOVA with Dunnett’s multiple comparisons test, with a single pooled variance. Data points represent values from each biological replicate. (D) Schematic of hcBBB conditions and infection procedure. WNV infection was performed 72 h after the initiation of co-culture. (E) WNV infection of the hcBBB system with only hBMECs, or hBMECs with hPSC-derived NCC-PCs or hPSC-derived astrocytes. The amount of infectious virus in the top and bottom chambers at 24 h post-infection was quantified by plaque assay. Bar graphs show the average from three independent experiments. Error bars show standard deviation. Conditions compared using a one-way ANOVA with Dunnett’s multiple comparisons test, with a single pooled variance. (F) Schematic of the no hBMEC hcBBB system used to measure apical (A) and basolateral (B) release of virus from hPSC-derived astrocytes, hPSC-derived NCC-PCs, or primary fibroblasts. Cells were plated on the Matrigel-coated underside of the semipermeable Transwell membrane. (G) The amount of infectious WNV released into the apical (A) and basolateral (B) chambers at 48 h post-infection was quantified by plaque assay. Bar graphs show the average value and standard deviation from three independent experiments. Apical and basolateral release for each cell type was compared using an unpaired *t* test.

BBB function is highly dependent on the interactions between the endothelial cells that make up the vascular wall and the perivascular cells that surround them. The generation and co-culture of multiple isogenic populations of BBB cells is a key advantage of hPSC-derived BBB models. The Transwell culture system consists of two culture compartments separated by a semipermeable membrane (Figure 3A). Previous hPSC-derived BBB models have cultured hPSC-derived BMECs on the semipermeable membrane and plated mural or brain cells in the lower chamber.^18^^,^^27^ This setup enables paracrine signaling between cell types but may not accurately recapitulate interactions that require close proximity between BMECs and pericytes or glial cells. *In vitro* BBB models using primary animal and human cells have shown that astrocytes and pericytes can be cultured on the basolateral side of the semipermeable membrane, generating a physiologically relevant model of BBB organization.^68^^,^^69^^,^^70^ To test whether we could generate a similar fully hPSC-derived BBB model, we cultured hPSC-derived pericytes or astrocytes on the basolateral side of Transwells seeded with hPSC-derived hBMECs (Figure 3A). Two days after hBMEC seeding, the insert was inverted, and Matrigel was added to the basolateral side of the membrane. Following Matrigel addition, hPSC-derived NCC-PCs or hPSC-derived astrocytes were added to the basolateral side of the membrane. Cells were allowed to adhere for 3 h, at which point the Transwell insert was placed back in a standard culture plate and media added to both compartments. To determine if our co-culture protocol negatively impacted hBMEC viability, we performed a fluorescence-based viability assay on hBMECs plated in the Transwell system. The assay discriminates between live and dead cells by simultaneously staining with green-fluorescent calcein-AM to indicate intracellular esterase activity and red-fluorescent ethidium homodimer-1 to indicate loss of plasma membrane integrity. The loss of green fluorescent signal in combination with red nuclei staining indicates cell death.

Viability was compared between hBMECs in the Transwell without undergoing the co-culture protocol and hBMECs after the co-culture protocol, which included a 30-min incubation with Matrigel and 3 3-h inverted incubation at 37°C. As a positive control, one set of hBMECs underwent the co-culture protocol and was then treated with 70% ethanol to induce cell death. Our results showed no increase in the number of dead hBMECs after undergoing the co-culture protocol (Figure 3B). The use of fluorescently tagged hPSC-derived NCC-PCs or astrocytes enabled easy confirmation of successful seeding of the basolateral side of the Transwell membrane by live cell imaging (Figure S6A). We refer to this hPSC-derived co-culture BBB model as the hcBBB model.

To identify the optimal co-culture conditions for our hcBBB model, we examined the effect of different media compositions in the basolateral chamber. We observed that prolonged astrocyte co-culture was significantly improved by replacing the media in the lower chamber with astrocyte media rather than sfPCM (Figure S6B). As our goal was to be able to culture both mural and glial cells on the basolateral side of the membrane using the same culture conditions, we confirmed that the NCC-PCs retained the expression of PC specific genes when cultured in astrocyte media. We examined the expression of the PC marker PDGFRb following five days of culture in either astrocyte media or sfPCM by immunofluorescence. Our results showed similar expression patterns in both conditions, suggesting that the NCC-PCs retained their cellular identity when cultured in astrocyte media (Figure S6C).

In agreement with previous reports showing that co-culture with pericytes or astrocytes can improve BMEC barrier function,^27^^,^^71^ the addition of either NCC-PCs or astrocytes to the basolateral side of the membrane resulted in increased TEER values across the hBMEC monolayer (Figure 3C). Critically, in the absence of hBMECs, neither NCC-PCs nor astrocytes showed TEER values above baseline, suggesting that the observed increase in TEER in the co-culture conditions was due to the influence of these cells on hBMECs. It should be noted that although the increase in TEER was statistically significant at 120 h after the initiation of co-culture, TEER values in all conditions decreased with prolonged time in culture, similar to observations in previous reports on the use of hPSC-derived BMECs.^55^^,^^72^

To test the utility of our hcBBB system in modeling viral infection at the BBB, we exposed the hcBBB system to WNV. In the hcBBB system, the apical side of the Transwell represents the blood, while the basolateral chamber represents the CNS. To determine if pericytes or astrocytes influence the release of virus at the BBB during viral infection, we added WNV to the apical side of the Transwell to initiate the infection of the hBMEC monolayer (Figure 3D). The addition of NCC-PCs or astrocytes did not impact the apical release of virus from the hBMEC monolayer; however, the addition of NCC-PCs to the system resulted in nearly a 10-fold decrease in the amount of virus present in the basolateral chamber relative to the hBMEC only control at 24 h post infection (Figure 3E). In agreement with the data presented in Figure 2B we did not observe a reduction in TEER due to WNV infection relative to mock infection in any of the conditions tested (Figure S7). There are conflicting reports on whether the WNV infection of BMECs in the presence of infected astrocytes reduces TEER values. Some studies have shown that matrix metalloproteinases secreted from WNV-infected astrocytes reduce endothelial cell barrier function,^64^ whereas other studies have shown that the WNV infection of BMECs cultured with astrocytes increases TEER values.^67^ In our studies, infection of the hcBBB system with either astrocytes or NCC-PCs resulted in a modest increase in TEER relative to mock infection.

In the hcBBB system, the virus is released apically and basolaterally from infected hBMECs, astrocytes, or NCC-PCs. To determine if infected NCC-PCs restricted basolateral release of virus relative to other cell types, we plated either NCC-PCs, astrocytes, or primary fibroblasts on the lower side of the membrane in the Transwell system and infected apically with WNV (Figure 3F). We quantified the amount of nascent virus released into the apical or basolateral chamber at 48 h post-infection. It should be noted that in this setup, no hBMECs were added to the apical side of the Transwell. The “No cell” condition is an empty Transwell with only Matrigel added on the underside of the membrane and is used to measure the amount of input virus remaining 48 h after infection. Our results showed that while astrocytes and fibroblasts showed no significant differences in the amount of virus released into the apical or basolateral chamber, NCC-PCs released significantly less virus into the basolateral chamber (Figure 3G). Collectively, this data suggests that NCC-PCs may possess a unique ability to restrict basolateral release of virus during infection at the BBB.

### Brain microvascular endothelial cells and pericytes display unique early transcriptional responses to West Nile virus blood-brain barrier infection

**Figure 4:**
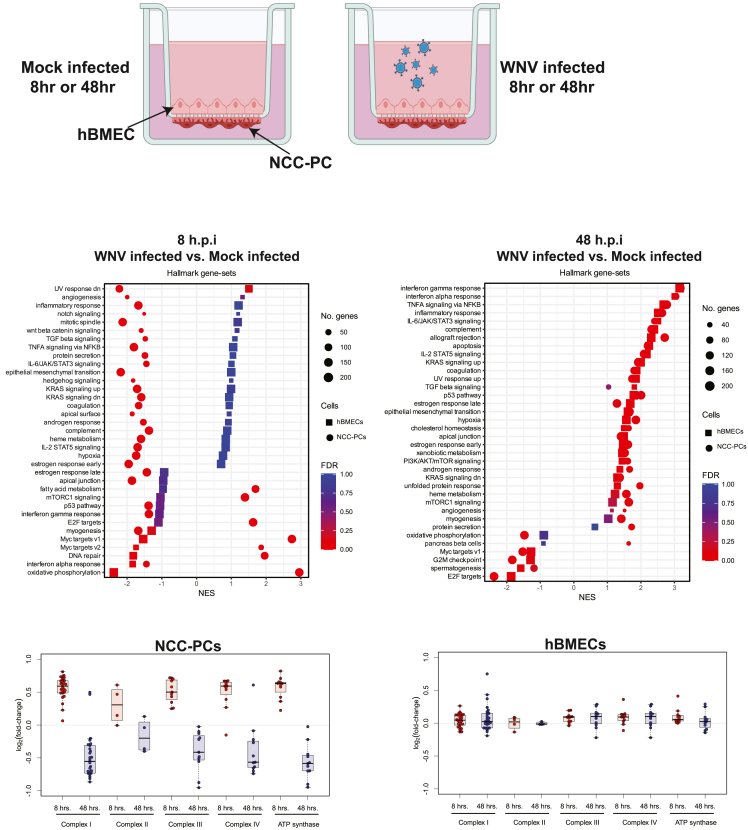
Transcriptional response to the infection of BMECs and NCC-PCs cultured in the hcBBB system (A) hPSC-derived BMEC-like cells (hBMECs) and NCC-PCs were plated in the hcBBB model as described in Figure 3A. WNV was added to the apical chamber at an MOI of 1. Total cellular RNA was isolated from hBMECs and NCC-PCs at the indicated time post-infection and analyzed by bulk RNA sequencing. (B, C) Gene set enrichment analysis (GSEA)^62^ was performed to compare WNV-infected versus mock-infected BMECs and WNV-infected versus mock-infected NCC-PCs at 8 and 48 h post-infection, respectively. The Hallmark collection^32^ of gene-sets from the MSigDB was used, and gene-sets were plotted only when an FDR of <0.05 for enrichment in at least one of the between-condition comparisons. (D, E) Average differential gene expression for genes that encode for proteins in the four complexes of the electron transport chain and ATP synthase. In the hcBBB model, log_2_(fold-change) was first estimated from RNA-seq data using DESeq2^73^ by comparing WNV-infected versus mock-infected NCC-PCs and WNV-infected versus mock-infected hBMECs, respectively, with statistical comparisons subsequently made over all genes in the indicated metabolic complex (see Table 1) using one-sample t testing.

When the circulating virus reaches the BBB, the first cells exposed to the virus are likely the BMECs that make up the vessel walls, followed by the mural or glial cells that surround these vessels. There is extensive cross-talk between cells at the BBB, which has the potential to influence an individual cell’s response to infection or influence cells that are in close proximity to infected cells. For this reason, analyzing the transcriptional profile of BBB cells in isolation likely does not provide an accurate picture of the response to infection. To gain insight into the transcriptional response of hBMECs and PCs at the BBB at discrete time points after exposure to WNV, we performed bulk RNA sequencing on hBMECs and NCC-PCs cultured in the hcBBB system following apical infection (Figure 4A). We examined the response at 8 h post-infection, a time point where we speculated that infection would not have spread from the hBMEC monolayer to NCC-PCs. We also examined the response at 48 h post-infection, a time point where we hypothesized that both cell populations would be infected. Critically, the gene expression patterns at each time point were compared to mock-infected hBMECs and NCC-PCs cultured in the hcBBB system, the observed changes in gene expression were likely not due to culture conditions but were instead a specific result of viral infection.

At both 8 and 48 h after virus exposure, the NCC-PCs exhibited very low levels of WNV infection relative to hBMECs. The fraction of normalized reads mapping to either the Env, NS1, or 3′UTR regions of the WNV genome was between 0 and 5% of the level for hBMECs (Figure S8A). Our data suggest that the hBMEC monolayer remains intact during infection (Figure S7), which supports the hypothesis that any virus that reaches the NCC-PCs must go through the hBMEC monolayer to reach the NCC-PCs. Thus, the viral infection of NCC-PCs is likely the result of either released progeny virions from infected hBMECs or transcytosis of input virus through the hBMECs.

In hBMECs at 8 h post infection, the overall changes in gene expression upon infection were small, with few cellular genes significantly different from mock-infected hBMECs (Figure S8B). Through gene set enrichment analysis (GSEA),^74^ we observed a downregulation in the IFN-α and oxidative phosphorylation gene-sets (Figure 4B). By 48 h post infection, both hBMECs and NCC-PCs showed strong upregulation of inflammatory signaling pathways, including IFN-α and IFN-γ responses and TNFα signaling (Figures 4C, S8D, and S8E). Interestingly, NCC-PCs showed nearly equivalent enrichment of inflammatory signaling pathways as hBMECs while having only about 20–25% as much viral RNA detected (Figure S8A). This result raises an intriguing possibility that NCC-PCs may be more sensitive to viral infection than hBMECs. However, as we do not have data with hBMECs at an equivalent infection level, as assayed by RNA content, we cannot conclusively compare the sensitivity of the antiviral responses. Combined, these results are consistent with a robust innate immune response to infection and suggest that during infection at the BBB, both BMECs and pericytes contribute to the local innate immune response.

At 8 h post-infection, NCC-PCs showed significant downregulation of a number of genes with a notable exception of HOXA1, which was significantly upregulated (Figure S8C). GSEA analysis on NCC-PCs 8 h after WNV exposure showed that the majority of gene-sets, including those involved in IFN-α and IFN-γ responses, were downregulated (Figure 4B). Surprisingly, the oxidative phosphorylation gene-set was strongly upregulated in NCC-PCs at 8 h post-infection. This result was particularly notable as the oxidative phosphorylation gene set was one of the most downregulated gene sets in the hBMECs at 8 h after virus exposure. To further explore the specific genes driving the upregulation of this gene set, we examined the differential expression of genes that produce the four large complexes that comprise the electron transport chain, as well as ATP synthase.^75^ We examined the expression of these genes in NCC-PCs at both 8 and 48 h after virus exposure to determine how expression levels changed over the course of infection. Our results show that the expression of genes encoding for Complexes I through Complex IV and ATP synthase is upregulated in NCC-PCs at 8 h post-infection relative to mock-infected NCC-PCs ($\mu_{\log_{2}\left(\text{fold}-\text{change}\right)}=0.53$, *p*-value = 3.7 × 10^−30^), whereas by 48 h post-infection the average expression of these genes is down in infected NCC-PCs relative to mock-infected NCC-PCs ($\mu_{\log_{2}\left(\text{fold}-\text{change}\right)}=-0.46$, *p*-value = 4.3 × 10^−15^, see Figure 4D; Table 1). Conversely, WNV infection of hBMECs resulted in much smaller changes in the expression level of these genes relative to mock infection (Figure 4E; Table 1), suggesting that the shift in the expression of metabolic genes may be a unique feature of NCC-PCs.

**Table 1:** Differential expression values of subunits of electron transport chain complexes

	NCC-PC 8 h.p.i	NCC-PC 48 h.p.i.	EC 8 h.p.i.	EC 48 h.p.i.
	Log2FC (WNV infected vs. Mock infected)			
**COMPLEX I**				

NDUFA3	0.73415583	−0.735119	0.12301695	0.43124366
NDUFS7	0.72121634	−0.7320411	0.05357259	0.0148358
NDUFA5	0.54199786	−0.2290166	−0.1336208	−0.0736978
NDUFV1	0.54765869	−0.4900453	0.16868784	−0.0190035
NDUFS8	0.66625103	−0.5322549	0.11984612	0.04564935
NDUFB1	0.81028164	−0.6963036	−0.0055606	0.23925238
NDUFS6	0.75361237	−0.7092134	0.11976456	−0.0074054
NDUFS2	0.52376423	−0.3101402	−0.0143121	−0.1924391
NDUFB7	0.70723726	−0.6308093	0.26131404	0.19612287
NDUFB5	0.43610581	−0.2854488	−0.1002111	0.07446164
NDUFC1	0.62065097	−0.8137267	−0.029226	0.173205
NDUFB4	0.62828558	−0.783272	0.05694262	0.00689658
NDUFV2	0.61541306	−1.5172208	0.12497301	0.36997966
NDUFA2	0.56648721	−0.6566156	0.08063125	−0.0740509
NDUFB2	0.73549161	−0.5732605	0.14714191	0.10586018
NDUFAB1	0.63370392	−0.3622364	0.14633021	0.02894066
NDUFA8	0.42065541	−0.4007474	0.1307616	−0.1004056
NDUFB6	0.675851	−0.7525585	−0.0845667	0.02123415
NDUFB3	0.67129141	−0.7048174	−0.0594731	0.1494488
NDUFA1	0.4980123	−0.4961723	0.08555226	0.14061784
NDUFC2	0.48693871	−0.4270126	−0.1318622	−0.009632
NDUFS3	0.32206844	−0.2984467	0.03398795	−0.0754295
NDUFA9	0.52576355	0.49739389	0.03638763	0.7485592
NDUFB8	0.48576734	−0.8646578	−0.0097366	−0.0729838
NDUFA7	0.22700329	−0.2027933	0.02679368	0.07615677
NDUFS1	0.06373369	0.46655682	−0.0638568	−0.1289948

**COMPLEX II**				

SDHD	0.46727013	−0.0399384	−0.1352324	−0.018834
SDHB	0.60830476	−0.3599651	0.08891354	0.02407335
SDHA	0.14562393	−0.4001886	0.05337868	−0.0127022
SDHC	−0.005411	0.1300804	−0.0164706	−0.0198931

**COMPLEX III**				

UQCR10	0.67070124	−0.5548368	−0.04022	0.12963891
UQCRC1	0.50050766	−0.5088408	0.1962969	0.13881084
UQCRB	0.71491723	−0.8746007	0.02915534	0.16153169
UQCRQ	0.69808171	−0.4813526	0.09859697	0.26074117
UQCRH	0.71845915	−0.9529868	0.19275587	−0.0257976
UQCRFS1	0.47317318	−0.2185043	−0.0125806	0.10068459
UQCRC2	0.34529886	−0.0252204	0.09490501	0.28639341
UQCR11	0.43217798	−0.413581	0.02507798	−0.07088
CYB5A	0.24890995	−0.1944255	0.07461507	0.07216987
CYB5R3	0.25683909	−0.0987683	0.11357212	−0.2211945
CYCS	0.57952169	−0.1280589	0.09055662	0.00647981

**COMPLEX IV**				

COX8A	0.66761701	−0.4698427	0.12606567	0.12963891
COX6C	0.6658216	−0.7407538	0.13326632	0.13881084
COX6B1	0.67176742	−0.5649744	0.09266763	0.16153169
COX5B	0.53852761	−0.2214017	0.14243971	0.26074117
COX5A	0.59532008	−0.5725552	−0.0174467	−0.0257976
COX6A1	0.59171239	−0.6355804	0.09178922	0.10068459
COX17	0.63230174	−0.6566795	0.35969916	0.28639341
COX7A2L	0.38849774	−0.088079	0.05144217	−0.07088
COX7C	0.57034503	−0.7001174	0.02693581	0.07216987
COX11	0.26717671	−0.2680604	−0.1120233	−0.2211945
COX10	−0.1521718	0.60814815	0.10663175	0.00647981

**ATP Synthase**				

ATP5MC2	0.57848386	−0.4800649	0.04341081	0.02250038
ATP5F1C	0.65546633	−0.6963364	0.09376902	−0.0025823
ATP5MC1	0.82062426	−0.9011945	0.05557779	−0.1123253
ATP5PD	0.61037096	−0.5723158	0.10155453	0.05430539
ATP5MC3	0.63220544	−0.4447375	0.01224289	−0.0308854
ATP5F1E	0.64426379	−0.6276007	0.12724126	0.28964142
ATP5PF	0.63426113	−0.6986329	0.02277773	0.04527836
ATP5MF	0.70758693	−0.5845765	0.18514455	0.24961349
ATP5F1B	0.35948702	−0.2202548	0.04488177	−0.146352
ATP5F1A	0.22225761	−0.0254319	−0.0003634	−0.0951949
ATP5F1D	0.42308395	−0.9463665	0.41109238	0.08927844

## Discussion

The BBB is a critical component of the innate immune system that safeguards the brain by preserving neural homeostasis, modulating neuroinflammation, and restricting viral invasion.^7^^,^^76^^,^^77^ Damage to the BBB is associated with cognitive impairment in both neurodegenerative disease and viral infection.^78^^,^^79^ Our data presented here show that pericytes may play critical roles in preventing both the disruption of the BBB and the spread of infection to the CNS.

In this study, we developed a serum-free protocol to produce neural crest-derived pericytes (NCC-PCs) from hPSCs and incorporated them into a fully hPSC-derived BBB model. Similar to previous models using primary cells,^68^^,^^69^^,^^70^ our model successfully mimics *in vivo* BBB anatomy, where brain microvascular endothelial cells and pericytes share a basement membrane, providing a physiologically relevant platform for studying BBB interactions and viral neuroinvasion.

It is well established that pericytes are essential for both the formation and maintenance of the BBB.^7^^,^^9^^,^^80^ Deficiency or dysfunction in pericytes results in increased BBB permeability and has been implicated in CNS pathologies, reinforcing a critical regulatory role in CNS function.^81^^,^^82^ We used our fully hPSC-derived BBB model (hcBBB) to investigate whether pericytes have a regulatory role when the circulating virus reaches the BBB. We show that pericyte exposure helps preserve barrier integrity in BMEC-like cells during POWV infection. In addition, incorporation of pericytes into the hcBBB system reduces WNV spread from the apical “blood” chamber to the basolateral “brain” chamber. Interestingly, our data show that although NCC-PCs are susceptible to viral infection, these cells are unique in that they restrict basolateral release of nascent virus. In future experiments, we hope to determine how pericytes achieve this unique regulation of viral release.

This study is the first to examine the transcriptional response of the cells that comprise the BBB to WNV infection in a physiological setting where paracrine signaling occurs between the cell types during infection. By promoting interactions between BMECs, pericytes, and astrocytes, our model provides a more accurate representation of the *in vivo* environment, allowing for a deeper understanding of how these cells collectively respond to viral invasion. In this model, the virus first infects BMECs and then spreads to NCC-derived pericytes. As a result, the infections are not temporally synchronized, making direct comparisons of the transcriptional responses between cell types at specific time points challenging. However, *in vivo*, BMECs and brain pericytes are also likely infected sequentially. Therefore, our goal with this model is to capture a snapshot of the transcriptional landscape in each cell type at different stages of viral spread across the BBB.

We observed that the transcription factor HOXA1 was significantly upregulated in pericytes early after WNV infection of neighboring hBMECs. HOXA1 has been linked to the *trans*-differentiation of vascular smooth muscle cells into macrophage-like cells.^83^ Pericytes have also been reported to adopt macrophage-like properties.^84^ It is possible that the upregulation of HOXA1 may promote this transition when the pericytes are exposed to nearby infected cells. This response, alongside metabolic changes, could represent a critical mechanism by which pericytes protect the brain during viral infection. While our studies focused primarily on the role of neural crest-derived pericytes at the BBB, it is possible that other mural cell types also play key roles in dampening the spread of infection at the BBB. Future studies incorporating smooth muscle cells and mesoderm-derived pericytes into the hcBBB system will test the cell-type-specific effects of brain mural cells during viral infection at the BBB.

Understanding how viral infections impact the metabolic state of an infected cell is an exciting area of research that may reveal novel therapeutic targets.^85^^,^^86^^,^^87^ We observed that early time points after infection, NCC-PCs in our hcBBB system increase the expression of genes involved in oxidative respiration. It has been proposed that pericytes switch to oxidative metabolism to promote terminal differentiation and restrict proliferation.^88^ Future studies are needed to investigate if changes in NCC-PC oxidative phosphorylation are protective or pathogenetic during viral infection at the BBB.

In conclusion, our study complements existing *in vivo* BBB models and provides a fully hPSC-derived system for modeling the interaction of cells at the BBB. In addition, our findings suggest that pericytes play an essential role in modulating viral infection at the BBB, providing new insights into how these cells can be leveraged for therapeutic interventions targeting viral neuroinvasion.

### Limitations of the study

Our study has several limitations. Although gene expression patterns suggest that our hPSC-derived NCC-PCs resemble brain pericytes, lower expression of markers such as NG2, CD13, and FoxF2 indicates they may be less mature than *in vivo* pericytes. As a result, the infection phenotypes observed in our BBB model may not fully reflect responses in mature pericytes. As noted earlier in the text, the hPSC-derived hBMECs used here likely contain a mix of epithelial and endothelial cells, which could influence their response to infection and interaction with pericytes. Future studies using hPSC-derived BMECs generated by alternative protocols will help validate these findings. WNV was selected for our initial experiments using the hcBBB system because of its lower biosafety requirements. However, the use of only WNV limits conclusions across other neurotropic flaviviruses; follow-up experiments with JEV and POWV are warranted. We also examined pericyte effects on BBB infection only at 24 or 48 hpi—longer time points are needed to assess durability and mechanism of protection. Finally, our findings on oxidative phosphorylation in NCC-PCs are based solely on transcriptional data; functional studies are needed to confirm metabolic changes.

## Resource availability

### Lead contact

Requests for further information and resources should be directed to and will be fulfilled by the lead contact, Dr. Rudolf Jaenisch (jeanisch@wi.mit.edu).

### Materials availability

This study did not generate new unique reagents.

### Data and code availability

• Data: Bulk RNA-seq data have been deposited at GEO at GSE302439 and are publicly available as of the date of publication.
• Code: All code used has been deposited at GEO at GSE302439 and is publicly available as of the date of publication.

## Acknowledgments

We acknowledge funding from the 10.13039/100016528Wyss Institute and the 10.13039/100019686Whitehead Institute. We thank the Genome Technology Core and Keck Imaging Facility at Whitehead Institute for Biomedical Research. This work was supported by 10.13039/100000002NIH grant U19AI131135, 10.13039/100000070NIBIB grant T32 EB016652. This work was performed in part in the 10.13039/100012802Ragon Institute BSL3 core, which is supported by the NIH-funded Harvard University Center for AIDS Research (P30 AI060354) and the 10.13039/100020366Massachusetts Consortium on Pathogen Readiness (10.13039/100020366MassCPR).

## Author contributions

A.R., A.K., and R.J. designed the project. T.W. and X.G. performed the bulk RNA sequencing data analysis. P.B. assisted with real-time PCR and portions of FACS data collection. L.G. facilitated work in the Ragone BSL3 facility. A.R. analyzed all data except for RNA sequencing data and prepared the article. All authors discussed the results and commented on the article.

## Declaration of interests

R.J. is an advisor/co-founder of Fate Therapeutics and Fulcrum Therapeutics. D.J.M. has sponsored research, consults, and/or has stock options/stock in Medicenna, Lyell, Attivare, Epoulosis, Limax Biosciences, Lightning Bio, and Oddity Tech, licensed intellectual property with Alkem and Amend Surgical, and Board of Directors, ATCC.

## STAR★Methods

### Key resources table

**Table:** 

REAGENT or RESOURCE	SOURCE	IDENTIFIER
**Antibodies**		

VE-Cadherin	R&D Systems	AF938
Calponin	Invitrogen	MA5-11620
NG2	Invitrogen	14-6504-82
Sox10	Cell Signaling	89356T
PDGFRβ	Cell Signaling	3169S
CD140b-PE	BD Pharmigen	558821
CD13-APC	BD Pharmigen	557454
Sox10- Alexa Fluor 488	Abcam	AB270150
Mouse-488	Life Technologies	A21202
Mouse-568	Life Technologies	A10037
Rabbit-488	Life Technologies	A21206
Rabbit-568	Life Technologies	A11011
Goat-488	Life Technologies	A11055

**Bacterial and virus strains**		

West Nile Virus (Nea Santa Greece 2010 Strain)	BEI Resources	NR-49928
Japanese Encephalitis Virus (India R53567)	BEI Resources	NR-2332
Powassan Virus (LB strain)	BEI Resources	NR-51181

**Chemicals, peptides, and recombinant proteins**		

CHIR 99021	Biogems	2520691
BMP4	Peprotech	120-05ET
EGF	Peprotech	AF-100-15
FGF-2	R&D Systems	BT-FGFB-GMP-025
Forskolin	Biogems	6652995
L-Ascorbic acid 2-phosphate sesquimagnesium salt hydrate (AA)	Sigma	A8960-5G
VEGF	Peprotech	100-20-50μg
SB 431542	Biogems	3014193
Heparin	StemCell Technologies	07980
PDGFbb	Peprotech	100-14B-10UG
CNTF	Peprotech	AF-450-13-500UG
Y-27632	Sigma	Y0503
Retinoic acid	Sigma	CH6H9A56EBC2

**Critical commercial assays**		

Live/Dead Viability/Cytoxicity Assay	Invitrogen	L3224
RNeasy Plus Mini kit	Qiagen	74004
SMART-Seq® v4 Ultra® Low Input RNA Kit	Takara Bio	634888
Swift RNA library prep kit	Swift Biosciences	R1024

**Deposited data**		

Raw and analyzed data	This paper	GEO: GSE302439

**Experimental models: Cell lines**		

IMR-90	ATCC	CCL-186
Vero E6	ATCC	CRL-1586
H1 embryonic stem cells (WAe001-A)	WiCell	WA01
iPS11 induced pluripotent stem cells	Alstem	iPS11

**Software and algorithms**		

FlowJo	BD Biosciences	https://www.flowjo.com/
Prism	GraphPad	https://www.graphpad.com/features
STAR aligner (v. 2.7.1a)	Dobin et al.^89^	https://github.com/alexdobin/STAR
featureCounts	Liao et al.^90^	https://rnnh.github.io/bioinfo-notebook/docs/featureCounts.html
DESeq2 (v. 1.36.0)	Love at al.^73^	https://bioconductor.org/packages/release/bioc/html/DESeq2.html

### Experimental model and study participant details

#### Human pluripotent stem cell lines and maintenance

H1 (WA01) embryonic stem cells were obtained from WiCell. iPS11 induced pluripotent stem cells were obtained from Alstem. All hPSCs were maintained in feeder-free conditions in mTeSR Plus media (StemCell Technologies) on Matrigel (Corning) in 6-well tissue culture dishes. For passaging, hPSCs were detached as clumps using Versene Solution (Thermo Fisher Scientific) and replated at a ratio of 1:8-1:10. For generation of fluorescently-tagged hPSC lines EGFP or tdTM was inserted into the *AAVS1* locus with a targeting plasmid (Addgene; catalog no. 22212) and a human codon-optimized SpCas9 and chimeric guide RNA expression plasmid (Addgene; catalog no. 42230), into which the Target Guide Sequence was cloned into using the oligos: Oligo 1: CACCGGGGGCCACTAGGGACAGGAT,Oligo2: AAACATCCTGTCCCTAGTGGCCCCC. After puromycin selection, EGFP or tdTM-expressing clones were manually picked. Clones homozygous for the insertion in the *AAVS1* locus were selected for further experimentation. All stem cell lines were routinely tested for mycoplasma contamination and found to be negative.

#### Ethics

All research presented in this manuscript complied with all relevant ethical regulations involving the use of human pluripotent stem cells and was approved by the Committee on the Use of Humans as Experimental Subjects (COUHES) and assigned IRB protocol number #0612002068.

#### Directed differentiation of hPSCs to neural crest-derived pericytes

H1 or iPS-11 hPSC were differentiated to Neural Crest cells using the Stemdiff Neural Crest Differentiation kit (Stem Cell Technologies Cat#08610) according to the manufacturer’s protocol. At passage two, neural crest cells were plated in Neural crest cell expansion media (NCC Expansion media, Table S1) on fibronectin coated plates. Neural crest cells were cryopreserved at passage three. For differentiation to neural crest-derived pericytes, neural crest cells were thawed directly into sfPCM (Table S1) onto fibronectin coated plates. Cells were cultured in sfPCM for a minimum of 10 days and passaged 1:3 when they reached 90% confluency. Between days 10 and 14, cells were cryopreserved at 200,000 cells/ml.

#### Directed differentiation of hPSCs to mesoderm-derived pericytes

All media recipes are described in Table S1. H1 hPSCs were cultured in E8 media (Thermo Fisher Scientific) and dissociated using Accutase (Thermo Fisher Scientific) into a single cell suspension and plated at 15,000 cells/cm^2^ in E8 media with Y-27632 (10 μM). Y-27632 was removed after 24 h, and cells were cultured in E8 media until they reached approximately 70% confluency, at which point the media was replaced with MelM media. This is considered day 0. On day 2, the media was replaced with PC/SMC1 media. On day 5, the media was replaced with PC/SMC2 media (for 24 h and then cells were passaged 1:3 onto fibronectin (FC01010MG, Fisher Scientific) coated tissue culture plates in PC3 media. Plates were coated with fibronectin at 10ug/ml for 30 min at 37°C prior to the addition of cells. After 48 h media was replaced with sfPCM media. After 48 h the media was replaced with fresh sfPCM and cells kept in culture for 7 days prior to freezing.

#### Directed differentiation of hPSCs to brain microvascular endothelial cells (BMECs)

The differentiation of H1 hPSCs to brain microvascular endothelial cells was based on a previously published protocols.^56^^,^^72^^,^^91^ Briefly, hPSCs were dissociated using Accutase (Thermo Fisher Scientific) into a single cell suspension and plated at 15,000 cells/cm^2^ in StemFlex media with Y-27632 (10 μM). Y-27632 was removed after 24 h and at 48 h post-plating the media was replaced with Unconditioned Media (UM) (100 mL Knock-out Serum Replacement (Thermo Fisher Scientific), 5 mL non-essential ammino acids (Thermo Fisher Scientific), 2.5 mL GlutaMax (Thermo Fisher Scientific), 5 mL Pen/Strep (Thermo Fisher Scientific), 3.5 μL β-mercapto-ethanol (Sigma), and 392.5 mL DMEM/F12(1:1), this was considered day zero. The media was replaced daily with fresh UM. On day six the media was changed to hESFM (Thermo Fisher Scientific) supplemented with 20 ng/mL bFGF (Peprotech), 10 μM retinoic acid (RA) (Sigma), and 1:50 B27 (Thermo Fisher Scientific). The media was not changed for 48 h. On day eight, cells were washed once with DPBS and incubated with Accutase for 30 min at 37°C. Cells were collected via centrifugation and plated onto either standard tissue culture plates or Transwell plates (Corning #3460). Both plates and Transwells were coated with 400 μg/mL collagen IV (Sigma Aldrich) and 100 μg/mL fibronectin (Fisher Scientific) overnight with collagen and fibronectin and washed 1X with PBS prior to the addition of cells. For tissue culture plates, cells were plated at a density of 250,000 cells/cm^2^, whereas for Transwell plates, cells were plated at a density of 1.1 x 10^6^ cells/cm^2^ on the Transwell membrane. bFGF and RA were removed from the medium 24 h after plating.

#### Directed differentiation of hPSCs to astrocytes

Astrocytes were generated using a protocol described in Tomasello et al.^92^ Briefly, stem cells were plated at a density of 90,000 cells/10 cm dish on Matrigel coated plates in Astrocyte media which was modified from Muffat et al.^93^ Astrocyte media contains 50% Neurobasal (Life Technologies, 21103049), 50% DMEM/F12/HEPES (Thermo Fisher Scientific, 12400024), 1:100 BSA-V (7.5%) (Gibco, 15260-037), 1:100 GlutaMAX (Life Technologies, 35050061), 1:100 Sodium Pyruvate (Life Technologies, 11360070), 1:100 *N*-2 (Life Technologies, 17502048), 20 ng/mL CNTF (Peprotech, AF-450-13-500UG), and 1:100 Penicillin Streptomycin. Astrocytes were passaged with Accutase (StemCell Technologies, 07920) when confluent, and maturity was confirmed with expression of markers roughly 150 days from 1^st^ media change.

#### Cryopreservation of cells

Cells were pelleted by centrifugation and resuspended in Freezing media A (50% culture media/50% FBS). An equal volume of Freezing media B (80% FBS/20%DMSO) was then slowly added to cells and mixed before aliquoting in cryogenic vials. Cells were frozen at a concentration of 2e^5^-5e^5^ cells/ml.

#### Cell lines and primary cells

The IMR-90 line of female primary human fibroblasts was obtained from ATCC (CCL-186) and cultured in 1X DMEM (Corning, 10–013) + 10% FBS and Pen/Strep, and split with 0.05% Trypsin for passaging. Vero E6 cells (African green monkey kidney cells) were obtained from ATCC (CRL-1586) and maintained in DMEM +5% FBS and Pen/Strep and passaged with 0.05% Trypsin when confluent.

### Method details

#### Virus propagation and titration

WNV (Nea Santa Greece 2010 Strain), JEV (India R53567), and POWV (LB Strain) were obtained from BEI Resources. All three viruses were propagated in Vero E6 cells cultured in Dulbecco’s modified Eagle’s medium (DMEM) supplemented with 2% fetal calf serum (FCS), penicillin (50 U/mL), and streptomycin (50 mg/mL). Viral titers were determined in Vero E6 cells by plaque assay. Work with POWV and JEV was performed in the biosafety level 3 (BSL3) at the Ragone Institute (Cambridge, MA) following approved SOPs. Work with WNV was done in BSL2+ containment at Whitehead Institute. For infection of BMECs, NCC-PCs, or Astrocytes the cell culture media was removed and replaced with inoculating virus diluted in a minimum volume of culture media. The cells were placed at 37°C for 1 h at which point the inoculum was removed and replaced with sfPCM (NCC-PCs), hESFM+B72 (BMECs), or astrocyte media (astrocytes).

#### Cell seeding in the hcBBB system

hPSC-derived BMECs were plated in 12-well Transwells (Corning#3460) as described above. Seventy-two hours after FGF/RA removal, the inserts were removed from the 12-well plates and placed upside down onto the inverted lid of a Deep Well 6-well plate (Corning Cat#355467). 80ul of Matrigel was added to the lower side of the semipermeable membrane (opposite side as BMECs). The Transwell inserts were covered with the lid of the Deep Well 6-well plate to prevent evaporation and placed at 37°C for 30 min. After incubation, excess Matrigel was removed, and 1.5e5 hPSC-derived astrocytes or hPSC-derived NCC-PCs resuspended in 100ul astrocyte media were added to the Matrigel-coated side of the Transwell insert. The Transwell inserts were again covered with the lid of the Deep Well 6-well plate and placed at 37°C for 3 h to allow cells to adhere. After incubation, the Transwell inserts were placed back in 12-well plates, and 0.5 mL hESFM+B27 was added to the top chamber of the Transwell, and 1.5 mL of astrocyte media was added to the lower chamber. The media was replaced in the upper and lower chambers every 48 h and the Transwell plate was kept at 37°C until the time of infection.

#### Live/dead viability assay

For the Live/Dead viability/cytotoxicity assay (Invitrogen, L3224), no cells were added during the 3-h hcBBB incubation. Assay was performed 6 h after BMECs underwent the co-culture protocol. Assay was performed according to the manufacturer’s instructions. For a positive control, hBMECs plated in the Transwell system were treated with 70% ethanol for 30 min to induce cell death. Cells were imaged on a Nikon Eclipse Ti-U microscope with Nikon NIS-Elements v5.21.03 software.

#### Measurement of transendothelial electrical resistance (TEER)

Resistance was recorded using an EVOM ohmmeter with STX2 electrodes (World Precision Instruments). TEER values were presented as Ω × cm^2^ following the subtraction of either an unseeded Transwell or a Transwell with only Matrigel coated on the underside of the semipermeable membrane). TEER values were multiplied by 1.12 cm^2^ to account for the surface area. TEER was measured three independent times for each sample and from three separate Transwells for each experimental condition.

#### Flow cytometry

For live surface marker staining, cells were trypsinized with TrypLE Express for ∼5–7 or until cells detached. Cells were washed once with FACS staining buffer consisting of 2 mM EDTA and 0.5% BSA in PBS and centrifuged. Cells were then resuspended and incubated in 100ul FACS staining buffer with fluorophore-conjugated antibodies at the concentrations listed in Table S2 for 30 min on ice. Cells were then washed twice with FACS staining buffer and resuspended in 1 mL FACS staining buffer prior to analysis.

For Sox10 staining, cells were fixed with 2% paraformaldehyde for 10 min at room temperature (RT). Following fixation, cells were washed twice with PBS and then permeabilized for 10 min at room temperature in permeabilization buffer (0.1% Triton X-100 in PBS with 0.5% BSA). Cells were then washed once with FACS staining buffer and incubated with the Sox10 antibody (see Table S2) for 1 h at room temperature. Cells were then washed twice with FACS staining buffer and resuspended in 1 mL FACS staining buffer.

All stained cells were analyzed on a BD FACSAria II UV/Blue/Green/Red 4-laser flow cytometer. Cells were isolated as a population and then selected for singlets before setting gates to determine positive populations.

#### Immunofluorescence microscopy

Cells were fixed with 4% paraformaldehyde for 15 min at room temperature. Fixed cells were permeabilized for 15 min using 0.1% TX-100. Cells were then blocked with 3% BSA in PBS for 1 h at room temperature. Cells were incubated with primary antibodies (see S2) diluted in antibody buffer (0.01%TX-100 + 1%BSA in PBS) overnight at 4°C. Cells were washed with 0.05% Tween in PBS and incubated for 1 h at room temperature with secondary antibodies diluted in antibody buffer (see Table S2) diluted in antibody buffer. DAPI was added at 1:10,000 during secondary antibody incubation. Cells were imaged on a Nikon Eclipse Ti-U microscope with Nikon NIS-Elements v5.21.03 software.

#### Bulk RNA-sequencing

Prior to RNA extraction hBMECs and NCC-PCs were separately removed from the semipermeable Transwell membrane by scraping cells into a small volume of PBS. RNA was then extracted from cells using the RNeasy Plus Mini kit (QIAGEN) following the manufacturer’s protocol. For cell characterization samples (Figures 1 and S1–S3) libraries were prepared using the Swift RNA library prep kit (Swift Biosciences). For hcBBB sample sequencing (Figures 4 and S5) libraries were prepared using the SMART-Seq® v4 Ultra® Low Input RNA Kit. All samples were sequenced on a NovaSeq 6000 sequencer.

#### Analysis of gene expression

Paired-end reads (51x 51 bp) were mapped to a reference genome using the STAR aligner (v. 2.7.1a).^89^ The reference genome was a composite of the human genome (GRCh38) and that of the West Nile virus (Nea Santa Greece 2010, GenBank HQ537483.1). Counts for human protein-coding genes and lncRNAs (ENSEMBL release 93 annotations), together with the viral genes, were tabulated using featureCounts.^90^ Differential expression of mRNAs was assessed for pairwise contrasts between conditions using estimated fold-changes and the Wald statistic in DESeq2 (v. 1.36.0).^73^ From exploratory analysis, within-condition variance was observed to be relatively uniform across conditions, so per-gene dispersions were estimated using all conditions. Unless otherwise noted, empirical Bayes shrinkage^94^ was applied to fold-change estimates. Gene set enrichment analysis was carried out between pairs of conditions using GSEA^74^(v. 4.1.0) with counts that were normalized by DESeq2 and 1000 gene-set permutations were used to estimate *p*-values. Gene-sets that were tested for enrichment came from the Hallmark collection^95^ at the MSigDB.

#### Cell type characterization from bulk RNA sequencing and PCA

RNA sequencing reads were mapped to the human genome (GRCh38) using the STAR aligner (version 2.7.1a). The mapped reads were assigned to the human genes (ENSEMBL release 101 annotations) using featureCounts with the parameters ‘-p -s 2’, suitable for paired-end reversely-stranded libraries. Before using the ‘prcomp’ function in R (version 4.2.1) to perform PCA, the normTransform function of DESeq2 (version 1.36.0) was used to normalize, center, and log-transform (with a pseudocount of 1) the counts matrix.

Publicly available scRNA-seq data were leveraged to compare the transcriptomes of our hPSC-derived NCC-PCs with those of *in vivo* PCs. The tags-per-million (TPMs) were collected for five scRNA-seq samples^53^^,^^96^^,^^97^^,^^98^^,^^99^^,^^100^ of *in vivo* PCs. Median gene lengths and the DESeq2 function fpkm were used to estimate TPMs for our bulk RNA-seq dataset and replicates averages were taken across replicates to generate scatterplots comparing transcriptomes (see Figure S4).

### Quantification and statistical analysis

Prism 10 (GraphPad) was used for all statistical analyses, except for statistical analysis of the bulk RNA sequencing data. The statistical details for all experiments are provided in the corresponding figure legends. Details for the statistical analysis of bulk RNA sequencing data are provided in the method details section.
