# Supplementary material for: A human blood-brain barrier model reveals pericytes as critical regulators of viral neuroinvasion
Source: iScience. 2025 Dec 13;29(1):114443. doi: 10.1016/j.isci.2025.114443 (PMC12803845; doi:10.1016/j.isci.2025.114443)
Supplement: Document S1. Figures S1–S8 and Tables S1 and S2 [file mmc1.pdf]

iScience, Volume *29*

Supplemental information

A human blood-brain barrier model reveals pericytes as critical regulators of viral neuroinvasion

Alexsia Richards, Andrew Khalil, Punam Bisht, Troy W. Whitfield, Xinlei Gao, David Mooney, Lee Gehrke, and Rudolf Jaenisch

Figure S1

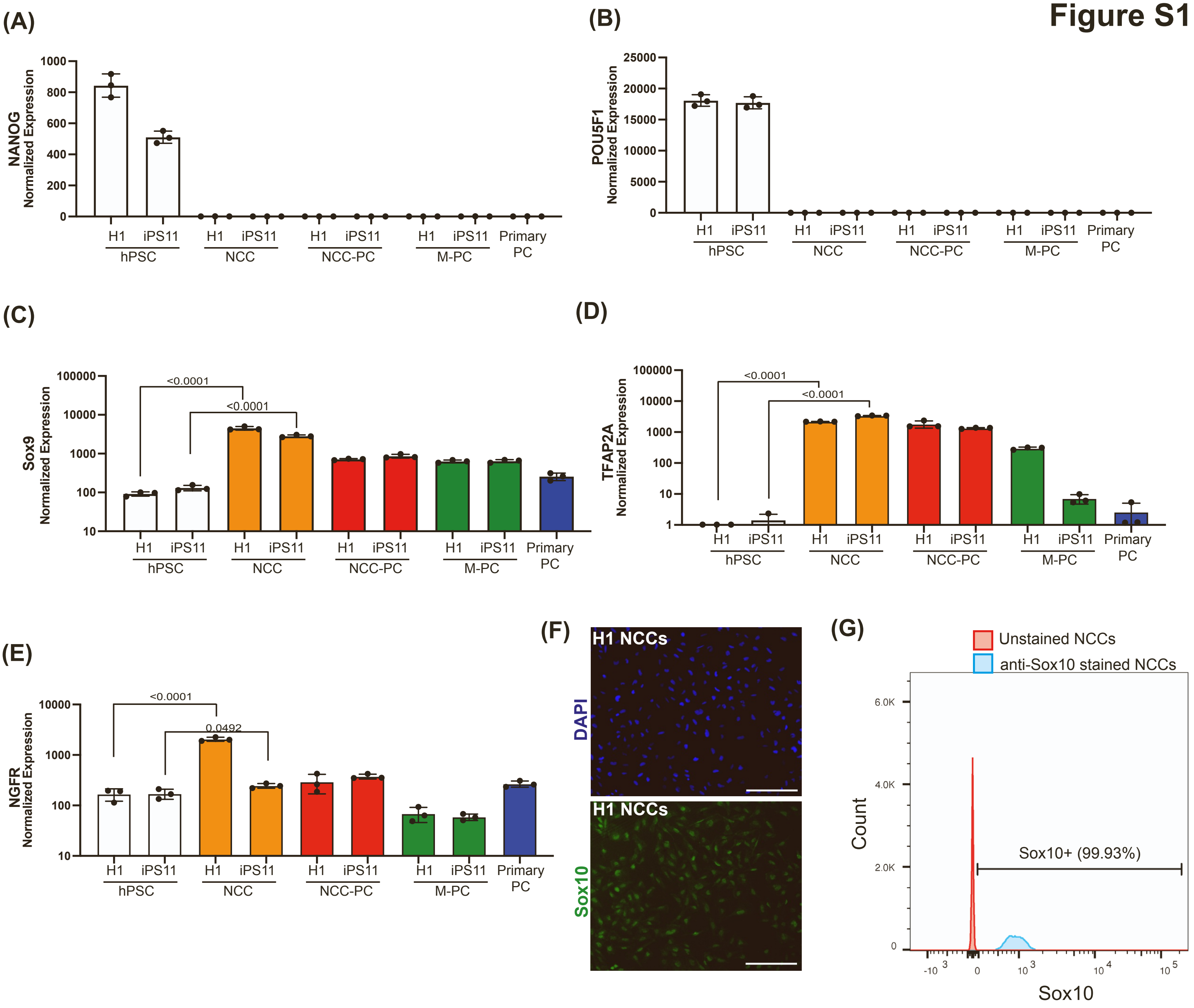

(A)
NANOG Normalized Expression
1000
800
600
400
200
0
H1
iPS11
hPSC
NCC
NCC-PC
M-PC
Primary PC
(B)
POU5F1 Normalized Expression
25000
20000
15000
10000
5000
0
(C)
Sox9 Normalized Expression
100000
10000
1000
100
10
<0.0001
<0.0001
(D)
TFAP2A Normalized Expression
1
(E)
NGFR Normalized Expression
0.0492
(F)
H1 NCCs
DAPI
Sox10
(G)
Unstained NCCs
anti-Sox10 stained NCCs
Count
6.0K
4.0K
2.0K
Sox10+ (99.93%)
Sox10

Figure S2

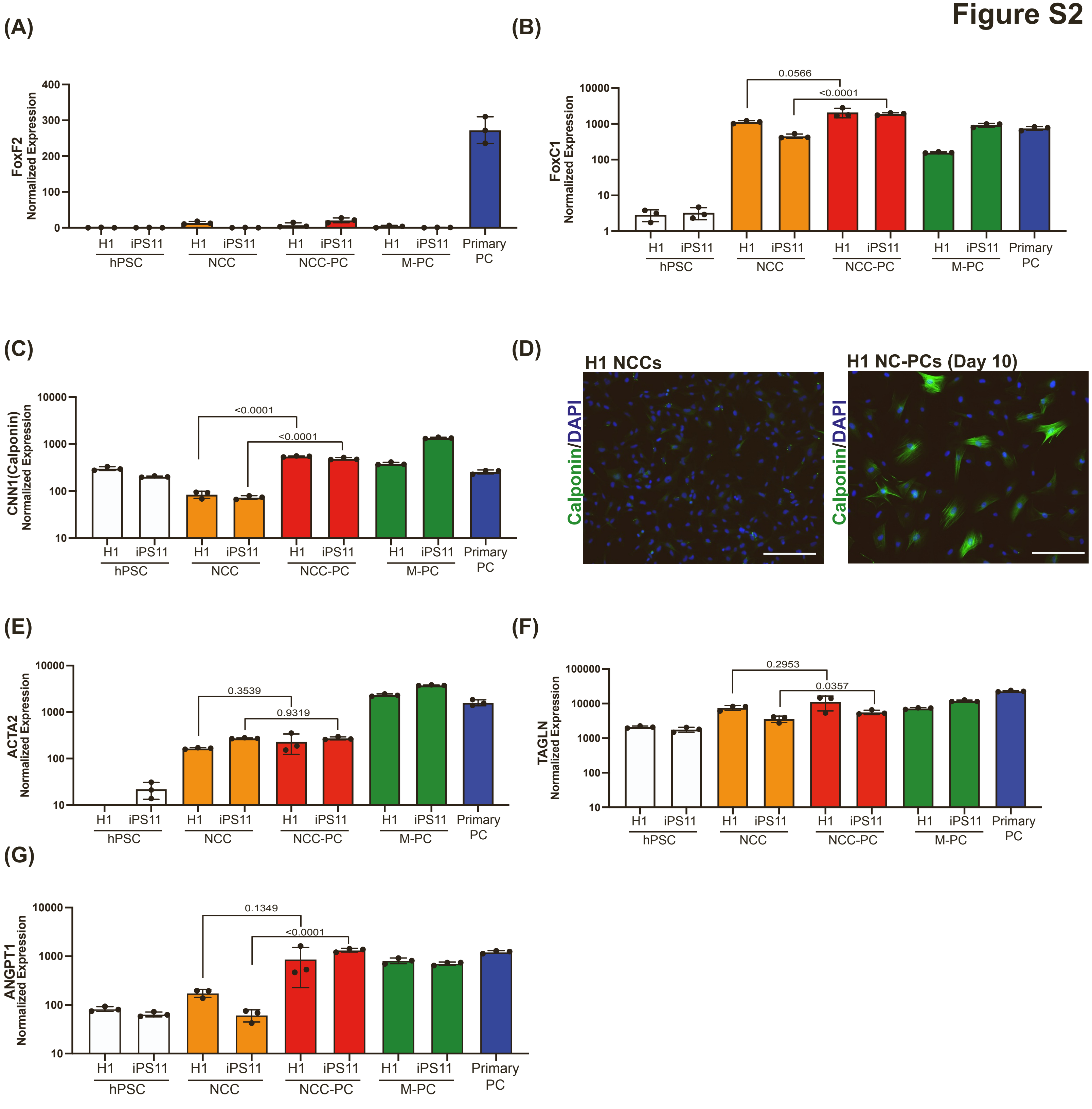

(A)
FoxF2 Normalized Expression
400
300
200
100
0
H1
iPS11
hPSC
NCC
NCC-PC
M-PC
Primary PC
(B)
FoxC1 Normalized Expression
10000
1000
100
10
1
0.0566
<0.0001
(C)
CNN1(Calponin) Normalized Expression
<0.0001
<0.0001
(D)
H1 NCCs
H1 NC-PCs (Day 10)
Calponin/DAPI
(E)
ACTA2 Normalized Expression
0.3539
0.9319
(F)
TAGLN Normalized Expression
100000
0.2953
0.0357
(G)
ANGPT1 Normalized Expression
0.1349
<0.0001

Figure S3

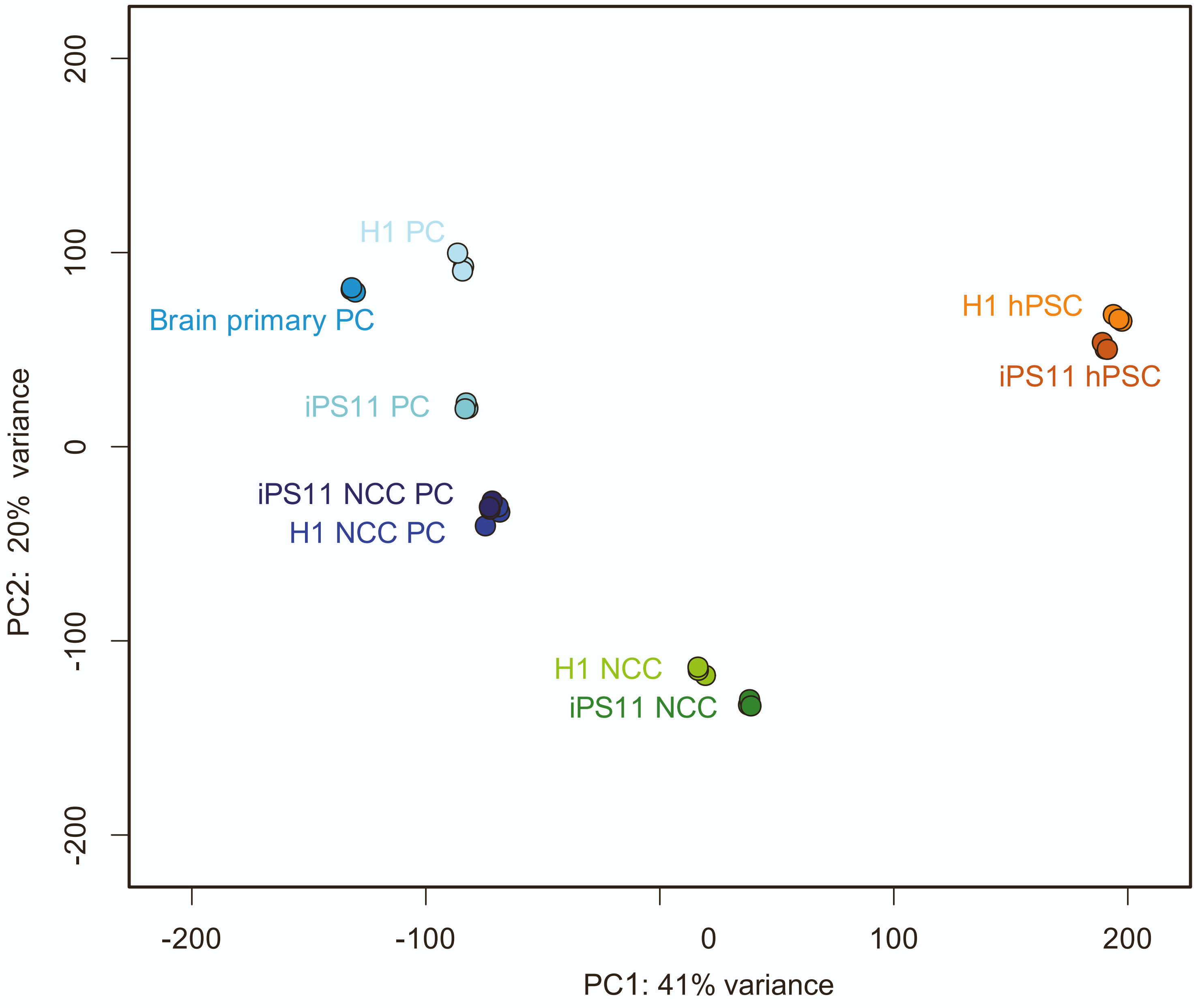

H1 PC
Brain primary PC
iPS11 PC
iPS11 NCC PC
H1 NCC PC
H1 hPSC
iPS11 hPSC
H1 NCC
iPS11 NCC
200
100
0
-100
-200
PC2: 20% variance
-200
-100
0
100
200
PC1: 41% variance

Figure S4

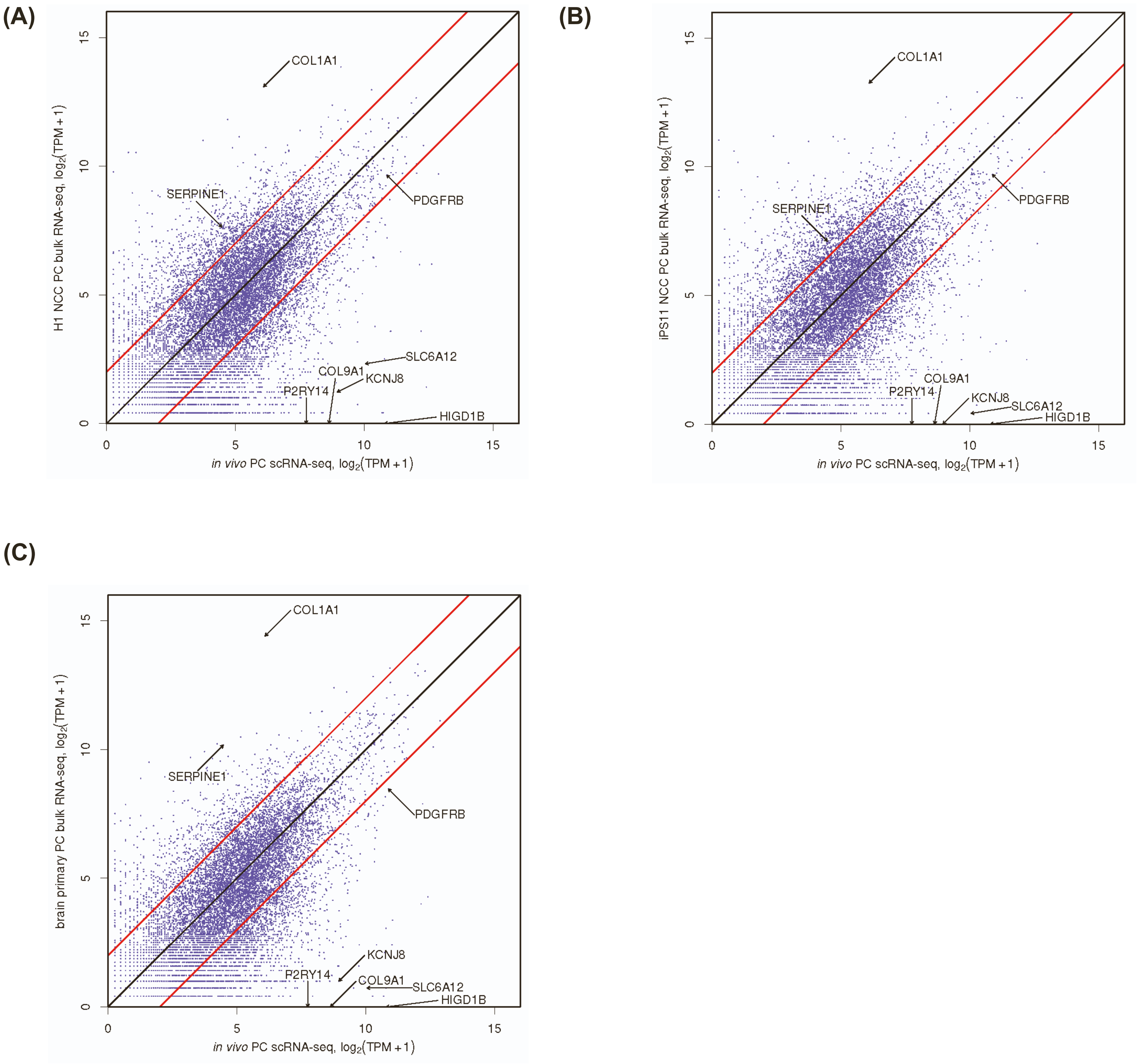
(A)
COL1A1
SERPINE1
PDGFRB
SLC6A12
COL9A1
KCNJ8
P2RY14
HIGD1B
H1 NCC PC bulk RNA-seq, log2(TPM + 1)
in vivo PC scRNA-seq, log2(TPM + 1)
0
5
10
15
(B)
COL1A1
SERPINE1
PDGFRB
COL9A1
P2RY14
KCNJ8
SLC6A12
HIGD1B
iPS11 NCC PC bulk RNA-seq, log2(TPM + 1)
in vivo PC scRNA-seq, log2(TPM + 1)
(C)
COL1A1
SERPINE1
PDGFRB
KCNJ8
P2RY14
COL9A1
SLC6A12
HIGD1B
brain primary PC bulk RNA-seq, log2(TPM + 1)
in vivo PC scRNA-seq, log2(TPM + 1)

Figure S5

(A)

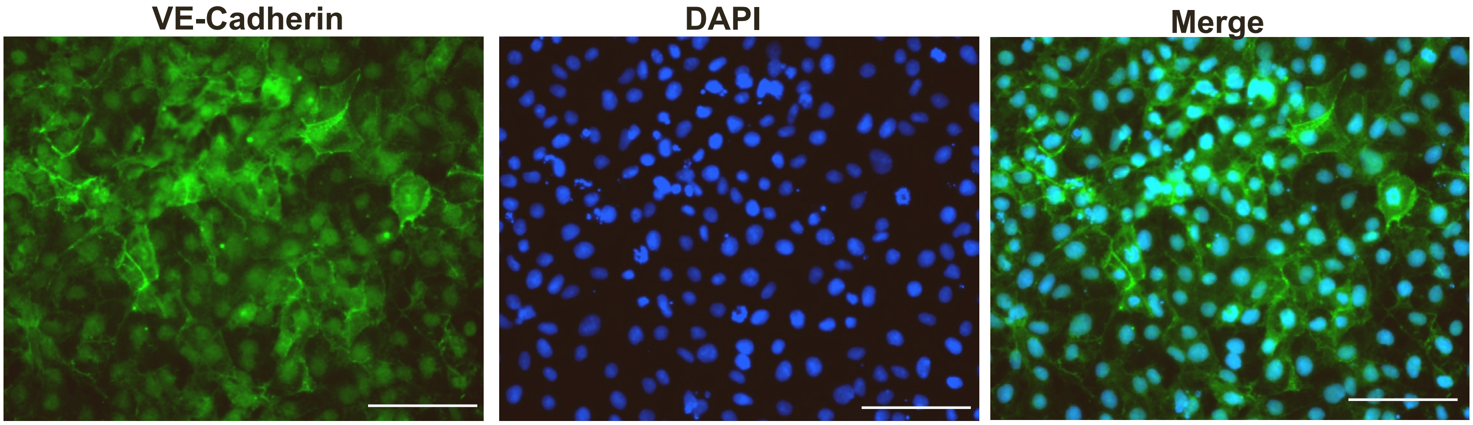
VE-Cadherin
DAPI
Merge

(B)

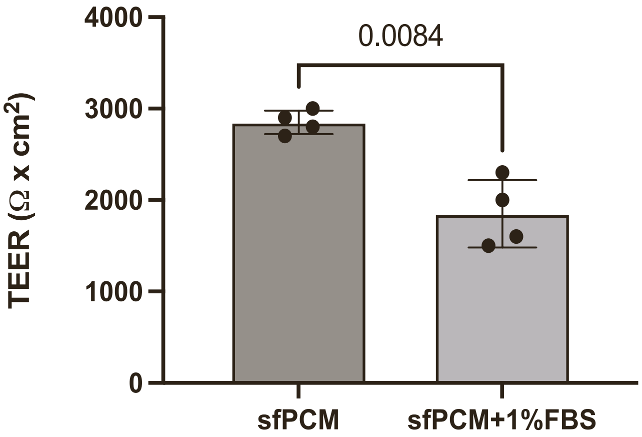
4000
3000
2000
1000
0
TEER (Ω x cm²)
0.0084
sfPCM
sfPCM+1%FBS

Figure S6

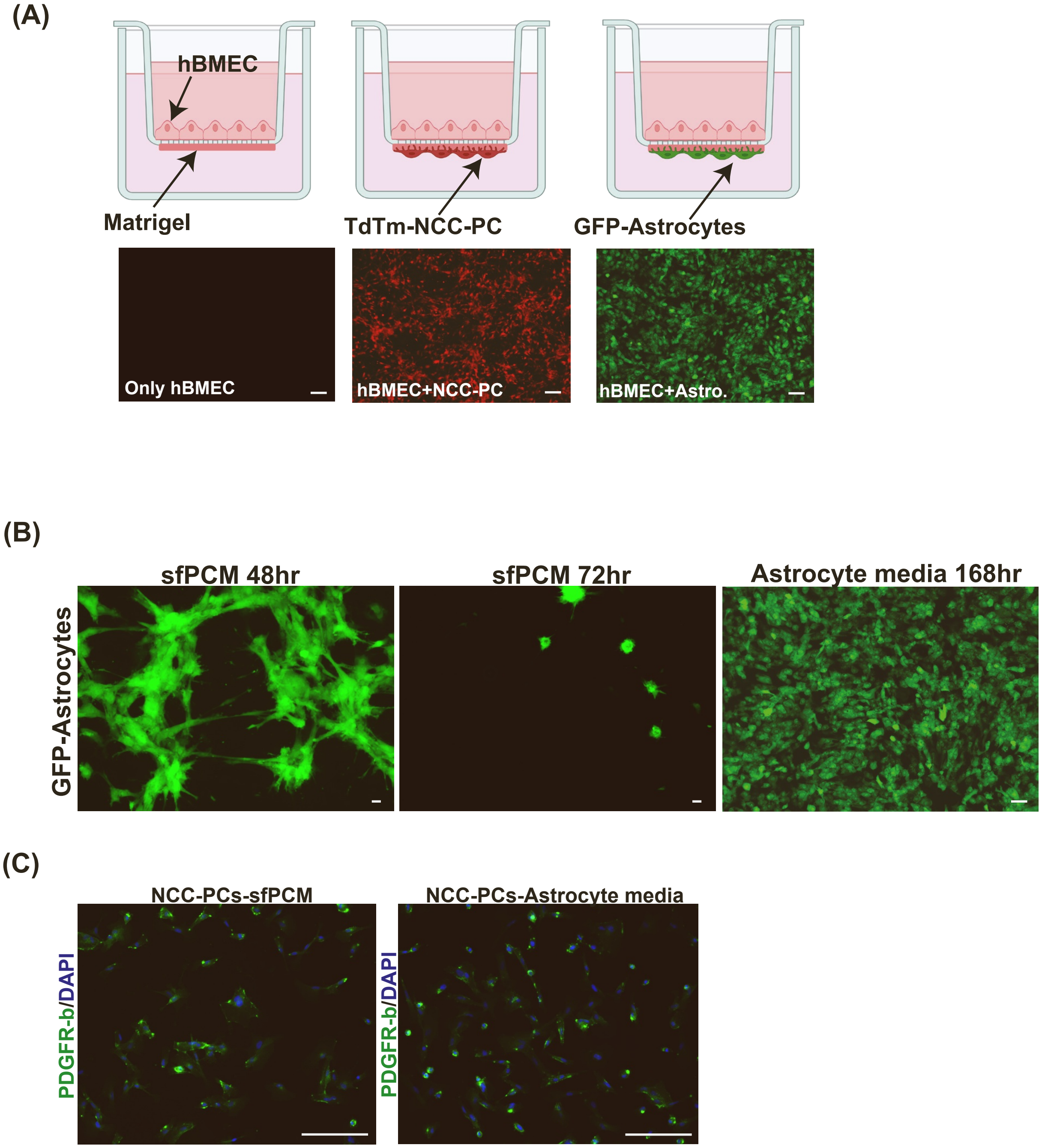

(A)
hBMEC
Matrigel
TdTm-NCC-PC
GFP-Astrocytes
Only hBMEC
hBMEC+NCC-PC
hBMEC+Astro.
(B)
sfPCM 48hr
sfPCM 72hr
Astrocyte media 168hr
GFP-Astrocytes
(C)
NCC-PCs-sfPCM
NCC-PCs-Astrocyte media
PDGFR-b/DAPI
PDGFR-b/DAPI

Figure S7

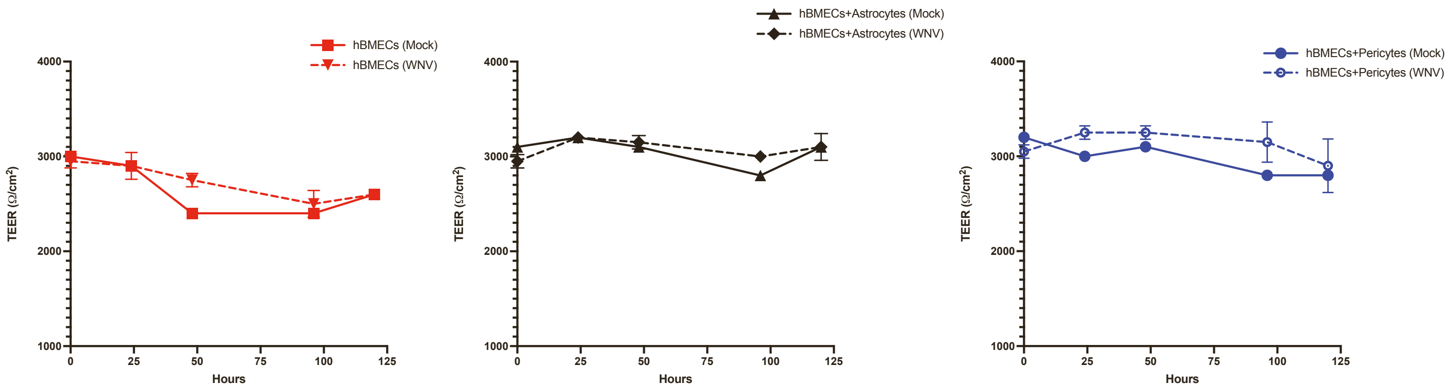

hBMECs (Mock)
hBMECs (WNV)
hBMECs+Astrocytes (Mock)
hBMECs+Astrocytes (WNV)
hBMECs+Pericytes (Mock)
hBMECs+Pericytes (WNV)
TEER (Ω/cm²)
Hours
4000
3000
2000
1000
0
25
50
75
100
125

Figure S8

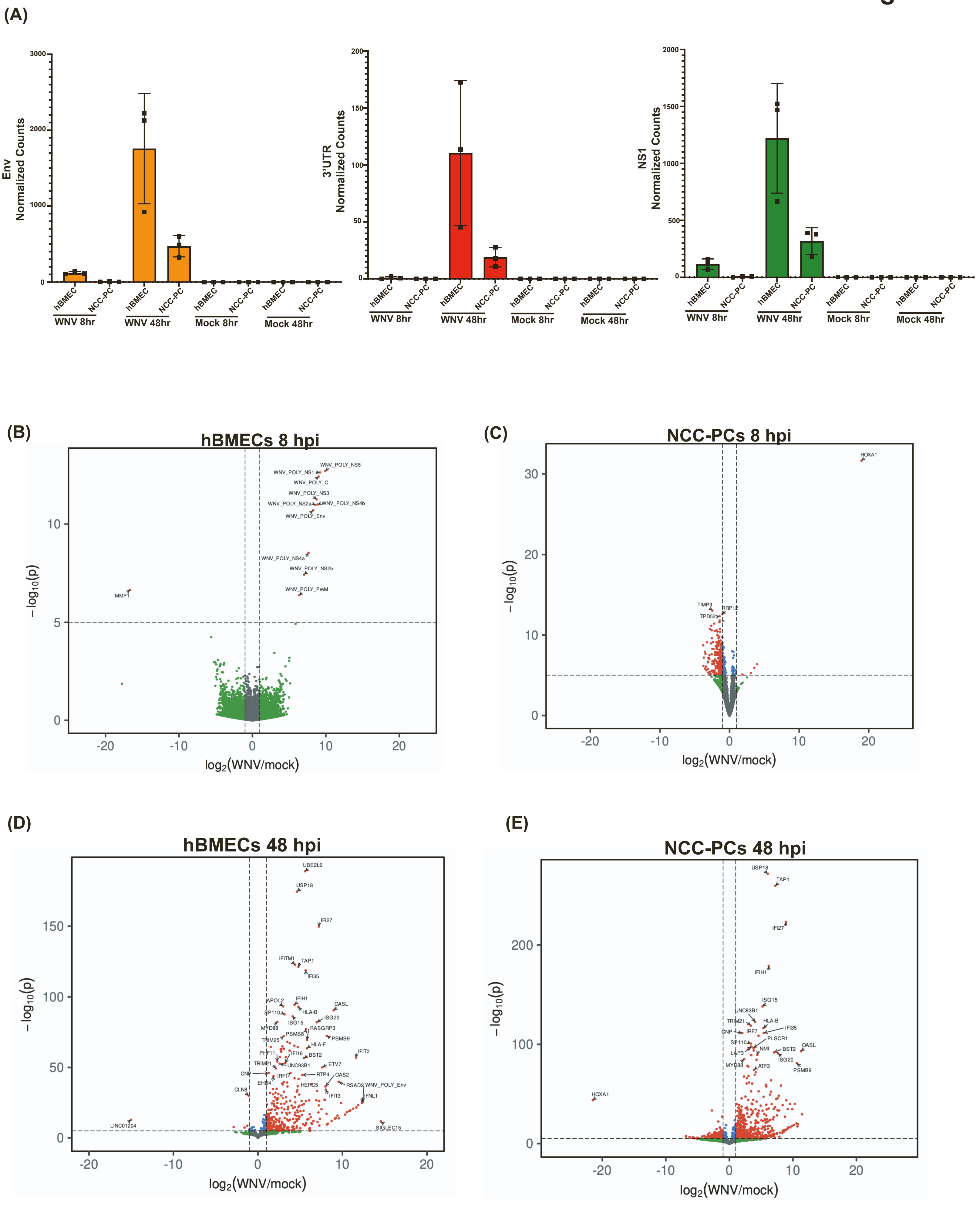
(A)
Env Normalized Counts
3'UTR Normalized Counts
NS1 Normalized Counts
hBMEC
NCC-PC
WNV 8hr
WNV 48hr
Mock 8hr
Mock 48hr
(B)
hBMECs 8 hpi
(C)
NCC-PCs 8 hpi
(D)
hBMECs 48 hpi
(E)
NCC-PCs 48 hpi
$-\log_{10}(p)$
$\log_2$(WNV/mock)

Supplemental Figure Legends

Figure S1: Characterization of hPSC-derived neural crest cells.

(A-E) Bulk RNA sequencing was performed on hPSCs, hPSC-derived neural crest cells (NCC), hPSC-derived NCC-PCs (NCC-PC), hPSC-derived mesoderm pericytes (M-PC), or primary CNS pericytes (Primary PC). H1 or iPS11 stem cells were used for all differentiations. All bar graphs show the mean value of three independent experiments, and error bars show the standard deviation. **(A)** Normalized RNA expression of NANOG **(B),** Normalized RNA expression of POU5F1 **(C),** Normalized RNA expression of Sox9 **(D),** Normalized RNA expression of TFAP2A **(E),** Normalized RNA expression of NGFR **(F).** Immunofluorescence images of H1 hPSC-derived NCCs stained with an antibody directed against Sox10. Scale bars =100um. **(G)** Expression of Sox10 quantified by flow cytometry in H1 NCCs. Fixed cells were stained with an Alexa 488-conjugated antibody directed against Sox10.

Figure S2: Characterization of hPSC-derived neural crest-derived pericytes.

(A-C, E-G) Bulk RNA sequencing was performed on hPSCs, hPSC-derived neural crest cells (NCC), hPSC-derived NCC-PCs (NCC-PC), hPSC-derived mesoderm pericytes (M-PC), or primary CNS pericytes (Primary PC). H1 or iPS11 stem cells were used for all differentiations. All bar graphs show the mean value of three independent experiments, and error bars show the standard deviation. **(A)** Normalized RNA expression of FoxF2 **(B),** Normalized RNA expression of FoxC1 **(C),** Normalized RNA expression of CNN1 **(D).** Immunofluorescence images of H1 hPSC-derived NCCs or H1 hPSC-derived NCCs-PCs

stained with an antibody directed against Calponin (CNN1). Scale bars =100um **(E)** Normalized RNA expression of ACTA2 **(F)** Normalized RNA expression of TAGLN **(F)** Normalized RNA expression of ANGPT1.

Figure S3: Comparison of hPSC-derived pericytes to cultured primary brain pericytes. Principal component analysis (PCA) on bulk-RNA sequencing data from hPSCs, hPSC-derived neural crest cells (NCC), hPSC-derived neural crest pericytes (NCC-PC), hPSC-derived mesoderm pericytes (PC), and primary brain vascular pericytes. Either H1 or iPS11 hPSCs were used for all differentiations as indicated in the figure.

Figure S4: Transcriptomic Comparison of hPSC-derived pericytes to *in vivo* brain pericytes. (A) Scatter plot comparing gene expression from single-cell RNA sequencing data on *in vivo* pericyte samples to bulk RNA-sequencing data from H1 NCC-PCs. Pearson's r = 0.78. Red lines indicate $\log_2$(fold-change) = 2 differences in gene expression between pairs of conditions. **(B)** Scatter plot comparing gene expression from single-cell RNA sequencing data on *in vivo* pericyte samples to bulk RNA-sequencing data from iPS11 NCC-PCs. Pearson's r = 0.77. Red lines indicate $\log_2$(fold-change) = 2 differences in gene expression between pairs of conditions. **(C)**Scatter plot comparing gene expression from single-cell RNA sequencing data on *in vivo* pericyte samples to bulk RNA-sequencing data from primary brain pericytes. Pearson's r = 0.80. Red lines indicate $\log_2$ (fold-change) = 2 differences in gene expression between pairs of conditions.

Figure S5: hPSC-derived BMEC-like cells express the endothelial marker VE-Cadherin and show reduced barrier function in the presence of serum. (A) Immunofluorescence images of H1 hPSC-derived BMECs (hBMECs) stained with an antibody directed against VE-Cadherin. Scale bar =100um**. (B)** hBMECs were plated on Transwell membranes, 48 hours after plating the media in the lower chamber of the Transwell was replaced with either sfPCM or sfPCM with 1%FBS. TEER was measured 48 hours after media replacement. Each data point represents an independent biological replicate. Bar graphs show the average value, and error bars show the standard deviation. Conditions were compared using an unpaired t-test.

Figure S6: Optimization of the hcBBB model. (A) Schematic of the generation of hPSC-derived co-culture blood-brain barrier model (hcBBB). Images show hPSC-derived tdTm-tagged NCC-PCs or hPSC-derived GFP-tagged astrocytes cultured on the underside of the Transwell semipermeable membrane in the hcBBB system. Scale bar=50um **(B)** hPSC-derived GFP-tagged astrocytes were cultured in the hcBBB system with either astrocyte media or sfPCM in the lower chamber. At the indicated time after the initiation of co-culture, astrocytes were imaged by fluorescence microscopy. Scale bar=50um (**C)** hPSC-derived NCC-PCs were cultured for five days in either sfPCM or astrocyte media and then fixed and stained with an antibody against PDGF-R beta. Scale bars=100um.

Figure S7: WNV infection of the hcBBB system does not result in loss of barrier function. The hcBBB system was established with either hBMECs only, hBMECs+ NCC-

PCs, or hBMECs+Astrocytes. WNV infection was performed 72 hours after the initiation of co-culture. TEER values were measured at the indicated time post-infection, each data point represents the average value of at least two independent biological replicates. Error bars show standard deviation.

Figure S8: NCC-PCs and BMECs in the hcBBB system display divergent transcriptional responses to WNV infection. hPSC-derived BMEC-like cells (hBMECs) and NCC-PCs were plated in the hcBBB model as described in **Fig. 4A**. WNV was added to the apical chamber at an MOI of 1. Total cellular RNA was isolated from hBMECs and NCC-PCs at the indicated time post-infection and analyzed by bulk RNA sequencing. **(A)** RNA-seq counts, normalized via the median-of-ratios method in DESeq2[88], for the Env, 3'UTR, and NS1 regions of the WNV reference genome following mock- or WNV-infection of the hcBBB model. Each data point represents a value from an independent biological replicate. Bar graphs show the average value, and error bars show the standard deviation. **(B)** Volcano plot showing differential gene expression in mock versus WNV-infected hBMECs at 8 hours post-infection. **(C)** Volcano plot showing differential gene expression in mock versus WNV-infected NCC-PCs at 8 hours post-infection. **(D)** Volcano plot showing differential gene expression in mock- versus WNV-infected hBMECs at 48 hours post-infection. **(E)** Volcano plot showing differential gene expression in mock- versus WNV-infected NCC-PCs at 48 hours post-infection. All volcano plots are shown without empirical Bayes shrinkage applied to fold-change estimates[89].

Table S1: Media formulations used for differentiation of hPSCs, Related to STAR Methods

	Final Concentration	Source	Catalog #
**MelM**			
E6		Thermo Fisher Scientific	A1516401
L-Ascorbic acid 2-phosphate sesquimagnesium salt hydrate (AA)	60ug/ml	Sigma	A8960-5G
CHIR 99021	8uM	Biogems	2520691
BMP4	25ng/ml	Peprotech	120-05ET
**NCC Expansion media**			
DMEM/F12		Thermo Fisher Scientific	11320033
B27	1:50	Thermo Fisher Scientific	17504044
N2	1:100	Thermo Fisher Scientific	17502048
L-glutamine	1:100	Thermo Fisher Scientific	A2916801
NEAA	1:100	Thermo Fisher Scientific	11140050
EGF	20ng/ml	Peprotech	AF-100-15
FGF-2	20ng/ml	R&D Systems	BT-FGFB-GMP-025
CHIR 99021	3uM	Biogems	2520691
**PC**			
E6		Thermo Fisher Scientific	A1516401
Forskolin	2nM	Biogems	6652995
AA	60ug/ml	Sigma	A8960-5G
VEGF	200ng/ml	Peprotech	100-20-50µg
**PC2**			
hESFM		Thermo Fisher Scientific	11111044
Forskolin	2nM	Biogems	6652995
AA	60ug/ml	Sigma	A8960-5G
VEGF	200ng/ml	Peprotech	100-20-50µg
**PC3**			
hESFM		Thermo Fisher Scientific	11111044
AA	60ug/ml	Sigma	A8960-5G
VEGF	200ng/ml	Peprotech	100-20-50µg
SB 431542	10uM	Biogems	3014193
**sfPCM**			
hESFM		Thermo Fisher Scientific	11111044
B27	1:50	Thermo Fisher Scientific	17504044
EGF	20ng/ml	Peprotech	AF-100-15
Heparin	2ug/ml	StemCell Technologies	07980
SB 431542	10uM	Biogems	3014193
PDGFbb	10ng/ml	Peprotech	100-14B-10UG

Table S2: Antibodies used in this study, Related to STAR Methods

**Primary Antibodies**	**Source**	**Catalog Number**	**Dilution**
VE-Cadherin	R&D Systems	AF938	1:250
Calponin	Invitrogen	MA5-11620	1:200
NG2	Invitrogen	14-6504-82	1:200
Sox10	Novus	NBP3-11395	1:2000
PDGFRβ	Cell Signaling	3169S	1:200
CD140b-PE (Flow)	BD Pharmigen	558821	1:10
CD13-APC (Flow)	BD Pharmigen	557454	1:10
Sox10- Alexa Fluor 488 (Flow)	Abcam	AB270150	1:25
**Secondary Antibodies**	**Source**	**Catalog Number**	**Dilution**
Mouse-488	Life Technologies	A21202	1:1000
Mouse-568	Life Technologies	A10037	1:1000
Rabbit-488	Life Technologies	A21206	1:1000
Rabbit-568	Life Technologies	A11011	1:1000
Goat-488	Life Technologies	A11055	1:1000
